# LATS1-modulated ZBTB20 perturbing cartilage matrix homeostasis contributes to early-stage osteoarthritis

**DOI:** 10.1038/s41413-025-00414-3

**Published:** 2025-03-12

**Authors:** Xue Hao, Jing Zhao, Liyuan Jia, Guangyu Ding, Xiaoju Liang, Fei Su, Shuai Yang, Yating Yang, Jing Fan, Weiping J. Zhang, Liu Yang, Qiang Jie

**Affiliations:** 1 Pediatric Hospital, Honghui Hospital, Xi’an Jiaotong University, Xi’an, 710054 China; 2 Xi’an Key Laboratory of Skeletal Developmental Deformity and Injury Repair, Xi’an, 710054 China; 3 Research Center for Skeletal Developmental Deformity and Injury Repair, School of Life Science and Medicine, Northwest University, Xi’an, 710069 China; 4 Institute of Orthopedic Surgery, Xijing Hospital, Fourth Military Medical University, Xi’an, 710032 China; 5 State Key Laboratory of Immunity and Inflammation, and Department of Pathophysiology, Naval Medical University, Shanghai, 200433 China

**Keywords:** Pathogenesis, Bone

## Abstract

Osteoarthritis (OA) is one of the most common degenerative joint diseases in the elderly, increasing in prevalence and posing a substantial socioeconomic challenge, while no disease-modifying treatments available. Better understanding of the early molecular events will benefit the early-stage diagnosis and clinical therapy. Here, we observed the nucleus accumulation of ZBTB20, a member of ZBTB-protein family, in the chondrocytes of early-stage OA. Chondrocytes-specific depletion of *Zbtb20* in adult mice attenuated DMM-induced OA progress, restored the balance of extracellular matrix anabolism and catabolism. The NF-κB signaling mediated disturbance of ECM maintenance by ZBTB20 requires its suppression of *Pten* and consequent PI3K-Akt signaling activation. Furthermore, the subcellular localization of ZBTB20 was modulated by the kinase LATS1. Independent approaches to modulating ZBTB20 via utilizing TRULI and DAPA can restore ECM homeostasis, improving the abnormal behavior and moderating cartilage degeneration. The compounds TRULI and DAPA modulating ZBTB20 may serve as anti-OA drugs.

## Introduction

Osteoarthritis (OA) is a common chronic joint disease, one of the leading causes of disability and pain worldwide, with pathologic changes including fibrillation and degradation of the articular cartilage, subchondral bone sclerosis, formation of osteophytes, as well as inflammation responses.^1^ The global prevalence of OA has exceeded 7% of individuals (528 million), with a continual rise due to the growing elderly demographics in recent years.^2^ Besides the impacts on health burden, OA significantly influences healthcare expenses and social care costs, with an average cost ranged from $700-$15 600 across the world.^3^ Currently, pharmacological treatments primarily focus on pain relief, while there is no approved disease-modifying therapy available for patients in the advanced stages, leading to the eventual necessity of joint replacement surgery.^4^ Therefore, identifying and defining the cellular and molecular cascade of events at early-stage OA will benefit the diagnose and treatment of OA at initial phases, ultimately facilitating the restoration of joint homeostasis.^5^

Both local and systemic inflammation has been proved to be included in risk factors of OA, contributing to both the initiation phase and development process, in a cascade of releasing the inflammatory cytokines, activation of associated signaling pathways, and subsequent enhanced degradation of the extracellular matrix (ECM).^5,6^ Therefore, continuously monitoring the cellular and molecular processes will aid in gaining a deeper comprehension of alterations occurring in early-stage OA chondrocytes. Thus, we established an in vitro time-course OA model via IL-1β stimulation, to map the transcriptome diagram in OA chondrocytes. Analysis combining ATAC-seq and RNA-seq revealed the potential involvement of ZBTB20, a member of the Zinc finger and BTB domain-containing protein family, in rapid response to inflammation stimuli of chondrocytes.

ZBTB20 is a ZBTB-containing transcription factor playing a critical role in multiple organ development including brain and liver, as well as physiological processes like regulating glucose and lipid metabolism, in a manner of suppressing the transcription level of target genes.^7–9^ In the developing skeleton of mouse, ZBTB20 regulates the terminal differentiation of hypertrophic chondrocytes via repression of *Sox9*,^10^ suggesting a potential role of ZBTB20 in articular cartilage chondrocytes, while its function in OA progression remains elusive.

In this study, we investigated the function and related mechanism of ZBTB20 in early-stage OA chondrocytes and identify therapeutic compounds that target ZBTB20. During the early-stage OA, ZBTB20 translocates into the nucleus but gradually diminishes as OA progresses. Chondrocyte-specific *Zbtb20* deletion reduces DMM-induced OA progression and restores ECM anabolic and catabolic balance. Mechanistically, ZBTB20 disrupts ECM maintenance through NF-κB signaling, requiring suppression of *Pten* and subsequent PI3K-Akt signaling activation. Additionally, the subcellular localization as well as transcription activity of ZBTB20 is regulated in a LATS1 dependent manner. Modulating ZBTB20 with TRULI and DAPA restores ECM homeostasis, alleviates OA progression, improves abnormal behavior, and moderates cartilage degeneration. By targeting the LATS1-ZBTB20 cascade with TRULI or DAPA, a new therapeutic strategy for OA management is proposed.

## Results

### Transcription factor ZBTB20 is activated in early-OA

**Fig. 1:**
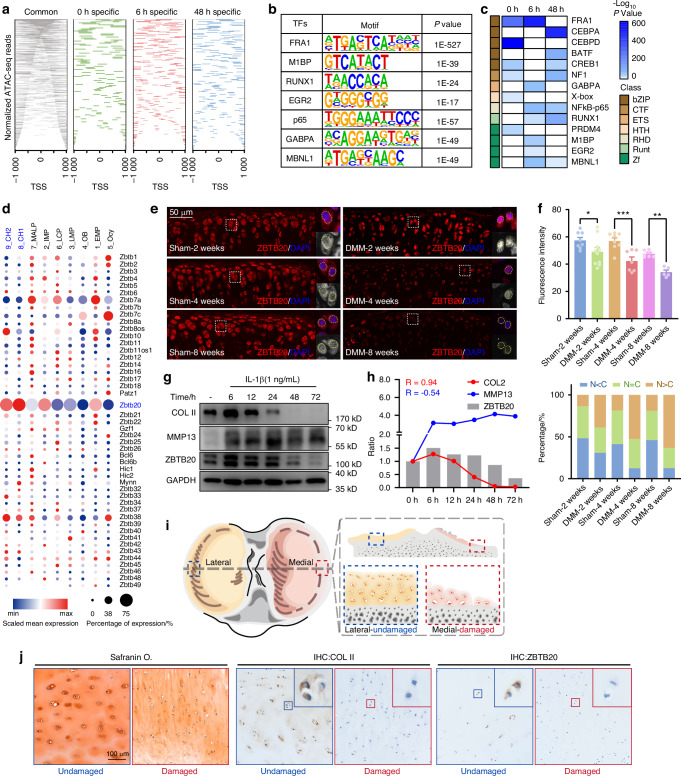
Transcription factor ZBTB20 is activated in early-OA. **a** Heatmap showing density of annotated ATAC-seq reads 1 kb around the transcription start sites (TSS). **b** Representative enriched TF binding motifs in chondrocytes treated by IL-1β for 6 h. **c** Heatmap of enriched motifs in chondrocytes under IL-1β stimulation for 0/6/48 h. **a**–**c** *n* = 3 replicates. **d** Dot plot graph showing the relative expression level and percentage of ZBTB-containing sub-family members in cell populations. **e** Representative images of immunofluorescence staining of ZBTB20 in articular cartilage of mice underwent DMM or Sham surgery for 2/4/8 weeks. Panels on the right are high magnifications of the boxed regions in the left panels. The dashed circles marked the edge of nuclei. *n* = 8, 10, 8, 7, 7, 5 mice. **f** Statistical analysis of the expressions of ZBTB20 in indicated groups of mice in (**e**). The top panel is quantification of relative fluorescence intensity of ZBTB20. The bottom panel is the statistical analysis for ZBTB20 cellular distribution. **g** Western blot of COL II, MMP13 and ZBTB20 in chondrocytes treated with IL-1β for 0-72 h. *n* = 3 biological independent experiments. **h** Curves of COL II, MMP13 and ZBTB20 expressions in (**g**). **i** Schematic diagram of cartilage tissues of the tibial plateau from patients. **j** Representative images of Safranin O/Fast Green and immunohistochemistry (IHC) staining for COL II and ZBTB20 in the relative damaged and undamaged cartilage from OA patients

To elucidate the biological processes of inflammation in osteoarthritis, an in vitro inflammatory model was established via subjecting the cultured primary chondrocytes to interleukin-1β (IL-1β), the most approved approach to mimic the process of inflammation.^11^ RNA-seq was performed on chondrocytes under IL-1β stimulation for 0/6/48 h, with 6 h considered as times point for rapid response and 48 h for long-term response. The DEGs were categorized into clusters according to their expression profiles. Besides the gradually upregulated or downregulated genes in C1, C3 and C4, a batch of genes exhibited elevated or inhibited expression especially for stimulation of 6 h, which enriched for biological process related to cytokines and cell cycle regulation (Fig. S1A). Subsequently, ATAC-seq was employed to investigate the chromatin accessibility profiling in chondrocytes (3 replicates for each, Fig. S1B). Consisting with the RNA-seq results, a gradual decrease was observed in regions near the gene promoter associated with extracellular matrix organization (*Acan*, *Col2α1*), while a significant increase was noted in genes relevant to immune responses (*Cxcl1*, *Cxcl2*, *Cxcl3*) (Fig. S1C). Analysis of TF binding motifs in each group indicated an enrichment of Zinc Finger-containing transcription factors in the 6 h samples, signifying the activation of ZF proteins (Figs. 1a–c and S1D). The higher proportion of down-regulating genes in 6 h compared to the 48 h samples suggested a suppressive role of these ZF transcription factors specifically activated at 6 h (Fig. S1E, F).

Among the ZF family, members of the ZBTB sub-family are mostly known for their transcriptional repressive functions.^12^ A single-cell sequencing outcome^13^ revealed that within the ZBTB-containing sub-family, ZBTB20 exhibits significant expression in clusters of chondrocytes (Fig. 1d). Given its role in modulating the inflammation response, we postulate that ZBTB20 could have a key role in the early pathogenesis of OA. Then we monitored the expression changes of ZBTB20 in OA progress. As shown in Fig. 1e and f, ZBTB20 in cartilage chondrocytes translocated into the nucleus at 2 weeks after DMM surgery, gradually diminished as OA advanced. In cultured chondrocytes, the protein level of ZBTB20 exhibited a downregulation trend upon IL-1β stimulation, correlated with COL II (Fig. 1g, h). To minimize variations in OA patients, cartilage samples from the lateral and medial regions of the tibial plateau were assessed as relatively damaged or undamaged areas. The reduction of ZBTB20 expression was observed in damaged region compared with undamaged region (Figs. 1i, S2 B–D). In conclusion, the integration of RNA-seq, ATAC-seq findings with the results of experiments on chondrocytes, mice, and clinical tissues indicates a strong association between the activation of ZBTB20 and the pathogenesis of OA in the early stages.

### Depletion of ZBTB20 attenuates OA progress

**Fig. 2:**
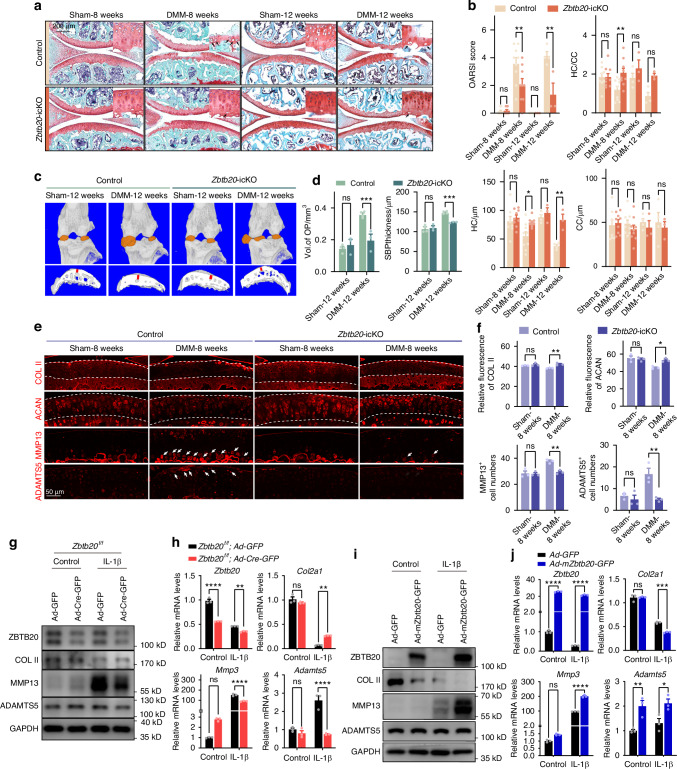
ZBTB20 icKO in chondrocytes alleviates OA. **a** Safranin O/Fast Green staining of articular cartilage from *Zbtb20*-icKO or corresponding control mice underwent DMM or Sham surgery for 8/12 weeks. Panels in the upper right corner are high magnifications of images. The arrows indicated the hypertrophy-like chondrocytes. *n* = 8, 9, 9, 9, 4, 3, 4, 3 mice. **b** Statistical analysis of OARSI scores, thickness of hyaline cartilage (HC), calcified cartilage (CC) and ratio of HC to CC in indicated groups of mice in (**a**). **c** Representative images of knee joint osteophytes (top panel) and subchondral bone plate (bottom panel) reconstructed by MicroCT analysis from indicated group of mice. *n* = 4, 3, 4, 3 mice. **d** Statistical analysis of volume of osteophytes (panel on the left) and SBP thickness (panel on the right) in indicated groups of mice in (**c**). **e** IF staining of COL II, ACAN, MMP13, and ADAMTS5 in the tibial cartilage from indicated group of mice. The dashed lines mark the edge of cartilage or the tidemark. The arrows mark the MMP13^+^ or ADAMTS5^+^ chondrocytes. **f** Statistical analysis of IF signal in indicated groups of cells in (**e**). *n* = 3, 3, 3, 3 mice. **g** Western blot of ZBTB20, COL II, MMP13, and ADAMTS5 in IL-1B treated *zbtb20*^*f/f*^ primary chondrocytes transduced with *Ad-GFP* or *Ad-Cre-GFP*. **h** Relative mRNA levels of *Zbtb20*, *Col2α1*, *Mmp3* and *Adamts5* in chondrocytes described in (**g**). **i** Western blot of ZBTB20, COL II, MMP13, and ADAMTS5 in IL-1B treated primary chondrocytes transfected with *Ad-GFP* or *Ad-mZbtb20-GFP*. **j** Relative mRNA levels of *Zbtb20*, *Col2α1*, *Mmp3*, and *Adamts5* in chondrocytes described in (**i**). **g**–**j** *n* = 3 biological independent experiments

Due to ZBTB20’s function of regulating hypertrophic chondrocyte differentiation in growth plate cartilage,^10^ we employed the TAM-inducible knockout strategy using *Col2a1-CreER*^*T2*^ mice to deplete ZBTB20 at 10 weeks old, to avoid the possible impacts from development defects of *Zbtb20*-deletion in chondrocyte. The DMM surgery to induce OA progress was carried out at 12 weeks old of mice, 2 weeks post the injection of TAM (Fig. S3A, B). OA manifestations observed previously included erosion of cartilage, increased ratio of hyaline to calcium cartilage thickness, formation of osteophyte, and heightened subchondral bone plate thickness.^14–16^ As shown in Fig. 2a and b, *Zbtb20* deficiency alleviated cartilage destruction post DMM-surgery at 8 and 12 weeks. The increased volume of osteophytes and thickness of subchondral bone plate were hindered by *Zbtb20* knockout (Fig. 2c, d). Hypertrophic differentiation has been reported to be one of the key factors promoting the initiation and progression of OA.^17^ As shown in Fig. 2a, the increased number of hypertrophic chondrocytes in articular cartilage was suppressed in *Zbtb20*-icKO mice. These findings highlight that *Zbtb20* depletion in chondrocytes decelerates OA progression.

The imbalance of ECM anabolism and catabolism is considered as the main cause of cartilage degradation, one of the most identical OA features.^18^ Therefore, the expression of proteins involved in ECM homeostasis was examined. The reduced expression of ECM components (COL II and ACAN) in OA cartilage was restored in Zbtb20 *icKO* mice, while the abnormal expression of ECM degradation enzymes (MMP13 and ADAMTS5) was blocked by *Zbtb20* knockout (Fig. 2e, f). In cultured chondrocytes, manipulation of *Zbtb20* expression through transduction of adenovirus or siRNAs, followed by IL-1β stimulation, resulted in similar outcomes as observed in vivo. Specifically, upregulation of MMP3, MMP13 and ADAMTS5 induced by IL-1β treatment was suppressed by *Zbtb20* knockout, and the downregulated expression of COL II was recovered in *Zbtb20* knockout chondrocytes (Figs. 2g, h and S4A–D). Moreover, overexpression of *Zbtb20* exacerbated the disordered ECM homeostasis, resulting in enhanced ECM degradation and impaired ECM synthesis (Figs. 2i, j and S4E). As shown in Fig. S1A, the NF-κB signaling is enriched in the DEGs specifically upregulated in chondrocytes treated by IL-1β for 6 h, when the translocation of ZBTB20 to nucleus is detected, highlighting the possible correlation of ZBTB20 and NF-κB signaling activation. Activation of this pathway was assessed by examining the expression of the target gene *Nos2* and the cellular distribution of p65. As shown in Fig. S5, *Zbtb20* deletion suppressed NF-κB signaling activation, and vice versa. Altogether, these findings collectively suggest that ZBTB20 accelerates OA progression by regulating ECM maintenance via triggering NF-κB signaling.

### Activation of NF-κB signaling by ZBTB20 requires suppression of *Pten*

**Fig. 3:**
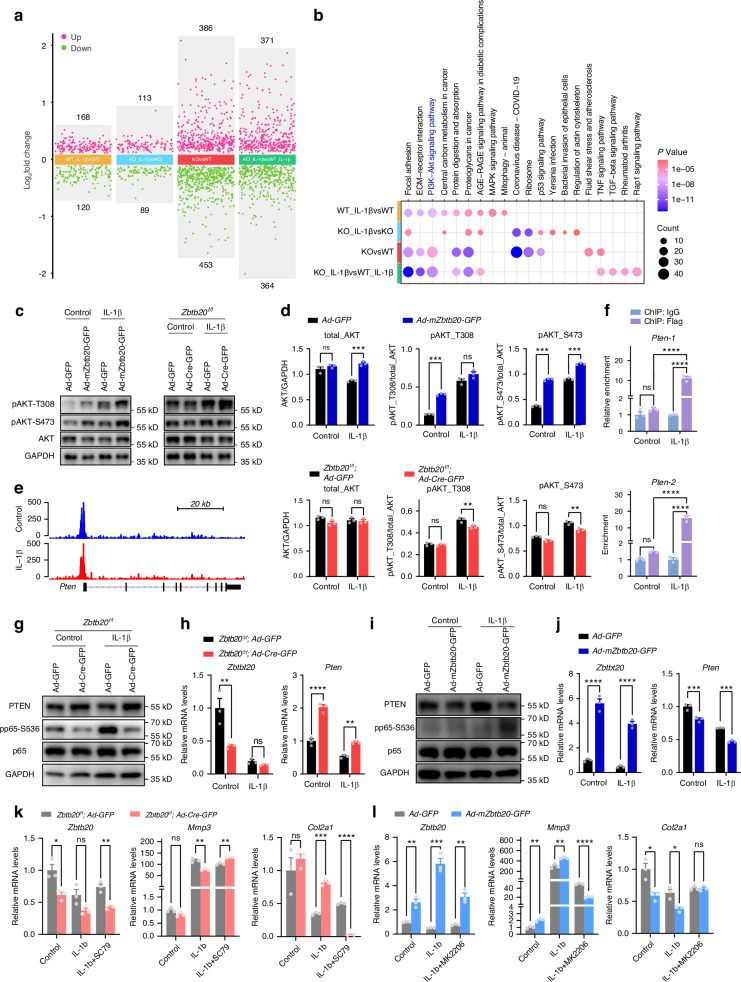
Activation of NF-κB signaling by ZBTB20 requires suppression of *Pten*. **a** Scatter plot graph showing the expression changes of DEGs from each comparison. **b** Dot plot graph of the top 10 enriched KEGG pathways of DEGs from each comparison. **a**, **b** *n* = 3 replicates. **c** Western blot of pAKT-T308, pAKT-S473, and AKT in indicated chondrocytes. **d** Statistical analysis of the bands in (**c**). **e** Representative tracks of CUT&Tag-seq analysis showing the enrichment of ZBTB20 around *Pten*’s promoter in chondrocytes. **f** Relative enrichment of ZBTB20 on binding regions compared to IgG in chondrocytes treated by IL-1β or not analyzed by ChIP-qPCR. **g** Western blot of PTEN, pp65-S536 and p65 in IL-1B treated *zbtb20*^*f/f*^ primary chondrocytes transduced with *Ad-GFP* or *Ad-Cre-GFP*. **h** Relative mRNA levels of *Zbtb20* and *Pten* in chondrocytes described in (**g**). **i** Western blot of PTEN, pp65-S536 and p65 IL-1B treated primary chondrocytes transduced with *Ad-GFP* or *Ad-mZbtb20-GFP*. **j** Relative mRNA levels of *Zbtb20* and *Pten* in chondrocytes described in (**i**). **k**, **l** Relative mRNA levels of *Zbtb20*, *Mmp3*, and *Col2α1* in indicated groups of chondrocytes treated with IL-1β or/and SC79/ MK2206. **c**–**l** *n* = 3 biological independent experiments

To elucidate the underlying mechanism of ZBTB20 triggering NF-κB signaling, RNA-seq analysis was carried out in WT and *Zbtb20* KO chondrocytes, brought up a series of DEGs through various comparisons (Fig. 3a). By integrating the top 10 enriched KEGG pathways of DEGs from each comparison, PI3K-Akt pathway emerged as a highly significant signaling pathways in ZBTB20-associated mechanisms, ranking high in WTvsWT_IL-1β group while not present in the KO_IL-1βvsKO group, with overlapping appearance in both KOvsWT and KO_IL-1βvsWT_IL-1β groups (Fig. 3b). The modulation of AKT phosphorylation by ZBTB20 reveals the involvement of PI3K-Akt signaling downstream of ZBTB20 indeed (Fig. 3c, d). To uncover the detail mechanism of ZBTB20 on PI3K-Akt signaling, CUT&Tag-seq^19^ analysis was performed in cultured chondrocytes that identified numerous peaks (Fig. S6A). Given the suppressive role of ZBTB20, the upregulated genes in KOvsWT comparison related to PI3K-Akt signaling, with ZBTB20 occupancy, were categorized based on their impact on signaling transduction, suggesting *Pten* as a crucial link between ZBTB20 and PI3K-Akt signaling (Fig. S6B). Distinct ZBTB20 peaks were detected in the promoter region of *Pten* in CUT&Tag-seq profiles, and further verified by ChIP-qPCR (Figs. 3e, f and S6C). Altered expressions of PTEN, inversely correlated with p65 phosphorylation, in *Zbtb20*-modulated chondrocytes further supported the suppressive role of ZBTB20 (Fig. 3g–j). Experimental validation using compounds targeting PI3K-Akt signaling was performed to confirm the mechanism. Activation of PI3K-Akt signaling by SC79 disrupted ECM homeostasis restored by *Zbtb20* knockout, whereas inhibition by MK2206 recovered the *Zbtb20* overexpression-induced imbalance (Fig. 3k, l). Taken together, these results indicate that the NF-κB signaling mediated disturbed ECM maintenance by ZBTB20 requires its suppression of *Pten* and consequent PI3K-Akt signaling activation.

### LATS1 regulates the cellular distribution of ZBTB20

Besides these detailed mechanistic findings, our hypothesis that ZBTB20 is activated at early-stage OA was assessed by examining the target genes’ transcriptome. A single-cell sequence analysis of OA clinical cartilage tissues revealed the transitions of proliferative chondrocytes (ProCs), prehypertrophic chondrocytes (preHTCs), and hypertrophic chondrocytes (HTCs) over the course of OA development, with preHTCs indicating an intermediate state between ProCs and HTCs.^20^ The dramatically reduced expression level of *PTEN* in preHTCs, as well as these ZBTB20-target-genes, specifically in preHTCs as opposed to other cells indicates the transcription activation of ZBTB20 in these preHTCs (Fig. S6D, E), further supporting our observations of the dynamic ZBTB20 cellular distribution change during OA progression.

**Fig. 4:**
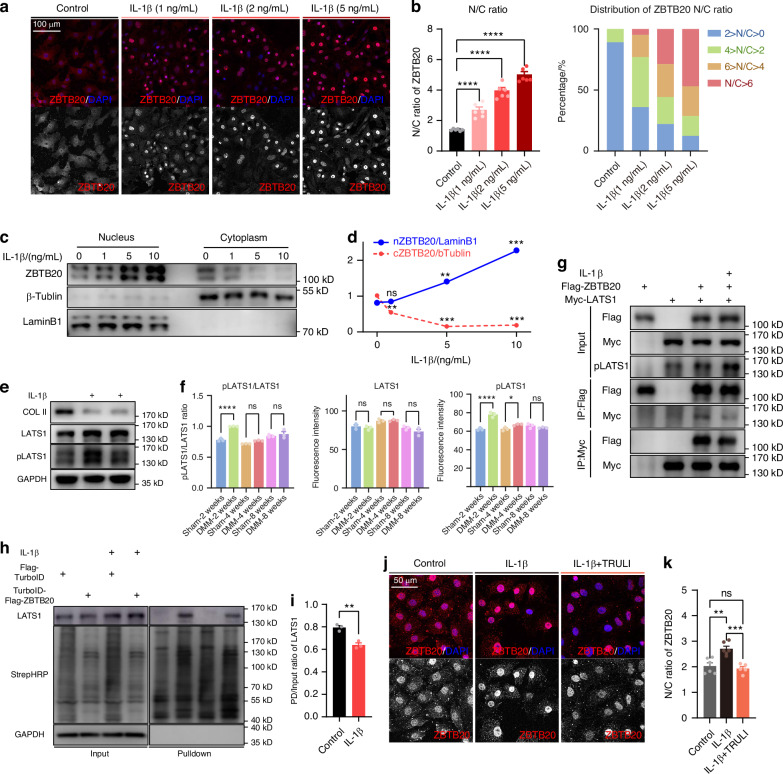
LATS1 regulates the cellular distribution of ZBTB20 in chondrocytes. **a** Representative images of immunofluorescence staining of ZBTB20 in primary chondrocytes exposed to IL-1β at concentrations ranging from 1 to 5 ng/mL. *n* = 3 biological independent experiments. **b** Statistical analysis of the cellular distribution of ZBTB20 in indicated groups of cells in (**a**). *n* = 6 views per group for one biological replicate. The left panel is quantification of N/C ratio of ZBTB20. The right panel is the statistical analysis for ZBTB20 cellular distribution. **c** Western blot of ZBTB20 in nucleus or cytoplasm extraction of chondrocytes treated with IL-1β ranging from 0 to 5 ng/mL. **d** Curves showing the cellular distribution of ZBTB20 in (**c**). **e** Western blot of COL II, LATS1 and pLATS1 in chondrocytes exposed to IL-1β. **f** Statistical analysis of fluorescence signal of LATS1 and pLATS1 in articular cartilage from mice underwent Sham or DMM surgery for 2/4/8 weeks. *n* = 3 mice per group. **g** Co-immunoprecipitation of Fg-ZBTB20 and Myc-LATS1. **h** Proximity labeling with TurboID and TurboID-ZBTB20 in chondrocytes. **i** Statistical analysis of bands of LATS1 in (**h**). **j** Representative images of immunofluorescence staining of ZBTB20 in primary chondrocytes upon IL-1β or/and TRULI stimulation. *n* = 3 biological independent experiments. **k** Statistical analysis of N/C ratio of ZBTB20 in (**j**). *n* = 6, 6, 5 views for one biological replicate. **c**–**e**, **g**–**i** *n* = 3 biological independent experiments

The increased nucleus accumulation of ZBTB20 was detected in chondrocytes upon IL-1β stimulation (Fig. 4a–d), validating our findings of ZBTB20’s activity change in OA pathogenesis, attributing to the ECM degradation via suppressing the expression of *Pten* and consequent NF-κB signaling activation at early stage of OA. Thus, approaches inhibiting ZBTB20 activity emerge as promising treatments for osteoarthritis. Since multiple challenges exist in modulating the activity of transcription factors, understanding the upstream regulation mechanism can provide insights for addressing these challenges, while the upstream regulating mechanism of ZBTB20 has been much less explored. The research conducted in *drosophila* revealed that the kinase Warts directly interacts with Lola, the homolog of ZBTB20 in *drosophila*, providing a clue to solve this question.^21^ Primarily, increased phosphorylated modification of LATS1 was observed in IL-1β treated chondrocytes as well as DMM-induced OA cartilage, especially 2 weeks after the surgery (Figs. 4e, f and S7A–C), indicating that the elevated phosphorylation of LATS1 may be the cause of ZBTB20 cellular re-distribution. Secondly, Co-IP was carried out using tag-fused proteins (Flag-ZBTB20, Myc-LATS1), turning out a reciprocal interaction of ZBTB20 and LATS1 to be detected, and weakened binding upon IL-1β stimulation (Fig. 4g). To further validate the interaction in situ, proximity labeling using TurboID-fused ZBTB20 was employed (TurboID-Flag-ZBTB20, Fig. S8). Consist with the Co-IP results, the interaction was proved by the detected biotin-labeled LATS1, suppressed by IL-1β treatment (Fig. 4h, i). Finally, supplementation of TRULI, a selective LATS1/2 inhibitor, countered IL-1β-induced nucleus accumulation of ZBTB20, indicating LATS1’s role in ZBTB20’s subcellular distribution (Fig. 4j–k). In summary, these results reveal that the kinase LATS1 controls cellular localization of ZBTB20. Phosphorylation of LATS1 induced by IL-1β treatment abolishes the interaction of ZBTB20 and LATS1, therefore releases ZBTB20 to translocate into nucleus.

As an essential element of the Hippo signaling pathway, a mechanical transduction singling, LATS1 undergoes phosphorylation and activation in response to mechanical stimuli.^22^ Since overloading is considered as one of the causes of OA, we wondered whether ectopic mechanical forces affect ZBTB20’s cellular distribution in chondrocytes in a LATS1-dependent manner. As shown in Fig. S9A–D, the nucleus accumulation of ZBTB20, as well as p65, is evident and reduced upon treatment with TRULI. This suggests that ectopic mechanical loading, which triggers LATS1 phosphorylation, in turn facilitates the translocation of ZBTB20 in chondrocytes.

### Both TRULI and DAPA restore ECM homeostasis in chondrocytes

**Fig. 5:**
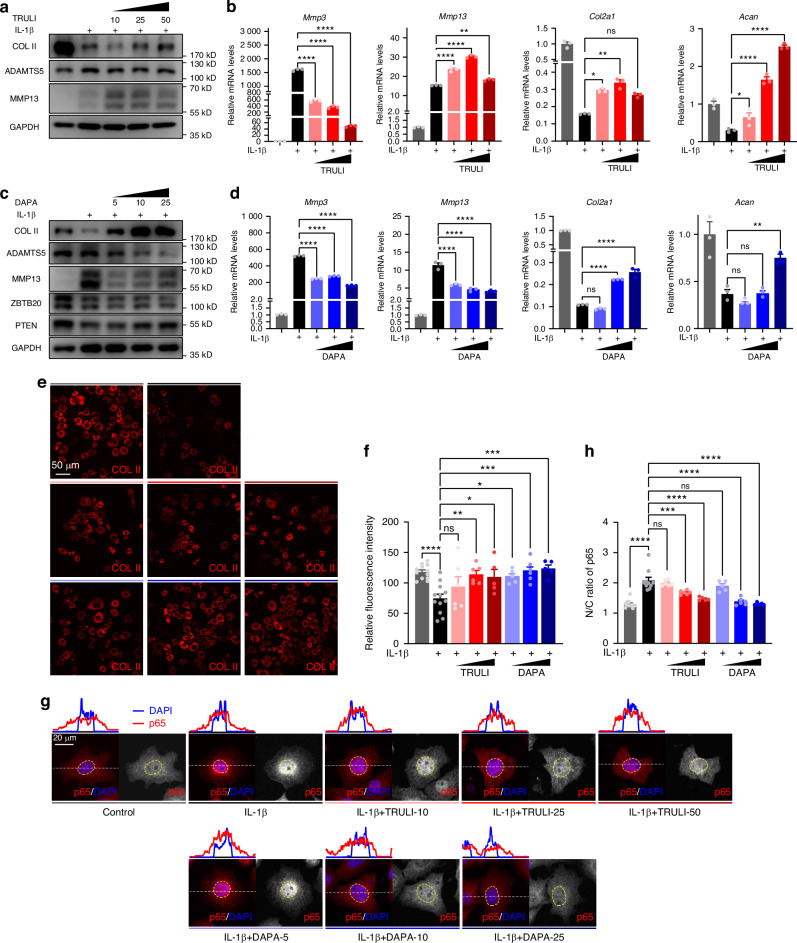
Both TRULI and DAPA are capable to restore ECM homeostasis. **a** Western blot of COL II, ADAMTS5, and MMP13 in chondrocytes treated with IL-1β, supplement with increased dosage of TRULI. **b** Relative mRNA levels of *Mmp3*, *Mmp13*, *Col2α1* and *Acan* in chondrocytes described in (**a**). **c** Western blot of COL II, ADAMTS5, and MMP13 in chondrocytes treated with IL-1β, supplement with increased dosage of DAPA. **d** Relative mRNA levels of *Mmp3*, *Mmp13*, *Col2α1* and *Acan* in chondrocytes described in (**c**). **e** Representative images of immunofluorescence staining of COL II in chondrocytes treated with IL-1β, supplement with increased dosage of TRULI or DAPA. **f** Statistical analysis of the relative fluorescence intensity of COL II in indicated groups of cells in (**e**). **g** Representative images of immunofluorescence staining of p65 in chondrocytes treated with IL-1β, supplement with increased dosage of TRULI or DAPA. Curves on the top of images showing the fluorescence intensity profile of crop section indicated by the white dashed line. The dashed circles mark the nucleus. **h** Statistical analysis of the cellular distribution of p65 in indicated groups of cells in (**g**). **a**–**e**, **g** *n* = 3 biological independent experiments. **f**, **h** *n* = 12, 12, 6, 6, 5, 5, 6, 5 views for one biological replicate

Our observations that in early-stage OA chondrocytes ZBTB20 translocates into the nucleus in a LATS1-dependent manner, triggers NF-κB signaling and enhances ECM degradation, leads us to consider different approaches to restore ECM balance: either by inhibiting LATS1 to impede ZBTB20 activity or by directly reducing ZBTB20 expression. As mentioned above, the small molecular compound TRULI can block the nucleus accumulation of ZBTB20 via inhibiting LATS1 phosphorylation. Then we evaluated the expressions of proteins associated with ECM homeostasis. The reduced ECM synthesis induced by IL-1β treatment was restored, while the abnormal ECM degradation was blocked by co-treatment of TRULI in a dosage dependent manner (Fig. 5a, b, e, f), indicating the therapeutic role of TRULI against OA. Research has identified *Zbtb20* as a potential therapeutic target of dapagliflozin (DAPA) from the RNA-seq analysis for the protective effects in hypertensive nephropathy,^23^ suggesting us DAPA as a proposing drug targeting *Zbtb20*. As shown in Fig. 5c, supplement of DAPA further minimized the expression of ZBTB20 indeed, as well as increased the expression of PTEN as a result. The disordered ECM balance, including disrupted expression of ECM components and ectopic expression of ECM degradation enzymes, was recovered by DAPA in a dosage dependent manner as well (Fig. 5c–f), suggesting DAPA’s protective effects in OA chondrocytes. Considering the crucial role of NF-κB signaling linking ZBTB20 and ECM maintenance previously described, its activation was assessed then. The increased nucleus accumulation of p65, reflecting the activation of NF-κB signaling, induced by IL-1β treatment was inhibited by both TRULI and DAPA gradually, further proving the protective role of TRULI and DAPA both (Fig. 5g, h). As described above, two independent approaches to modulating ZBTB20 via utilizing TRULI and DAPA can restore ECM homeostasis through triggering NF-κB signaling.

### Both TRULI and DAPA may serve as anti-OA drugs

**Fig. 6:**
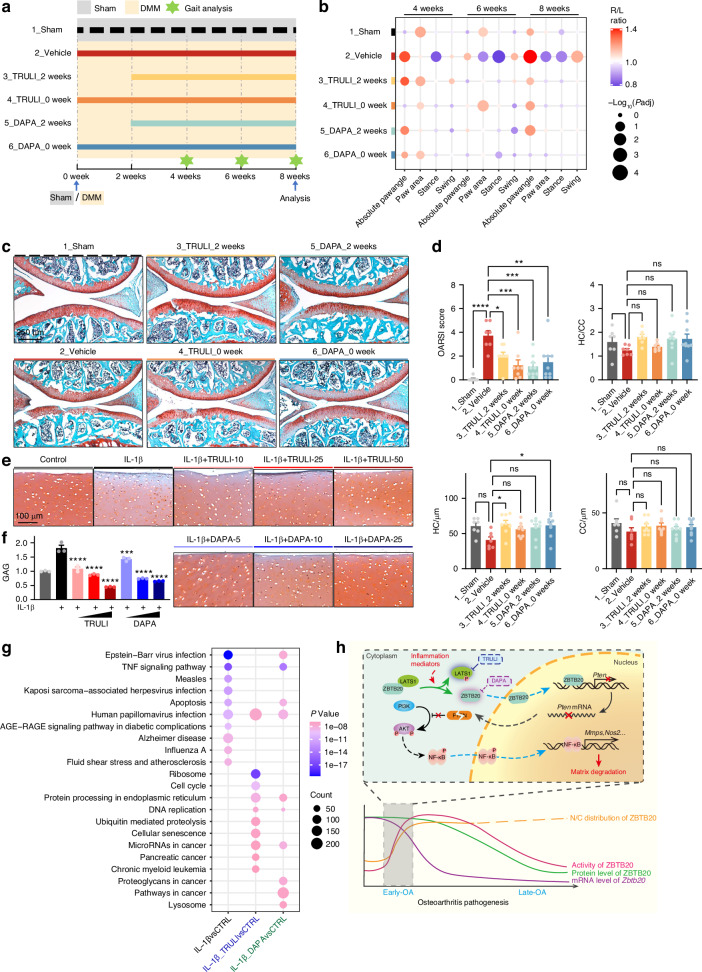
Both TRULI and DAPA may serve as anti-OA drugs. **a** Schematic diagram of administration of TRULI and DAPA in mice in injury induced OA model. **b** Dot plot graph showing the behavior abnormality indicated by ratios of the parameters between the right hind paw and left hind paw in each group of mice. **c** Representative images of Safranin O/Fast Green staining of articular cartilage from indicated groups of mice. **d** Statistical analysis of OARSI scores, thickness of hyaline cartilage (HC), calcified cartilage (CC) and ratio of thickness of HC to CC in indicated groups of mice described in (**a**). **a**–**d** *n* = 7, 7, 8, 8, 9, 9 mice. **e** Representative images of Safranin O/Fast Green staining of cultured cartilage explants from patient exposed to IL-1β, supplement with increased dosage of TRULI or DAPA. **f** Statistical analysis of released GAG into medium by DMMB assay in cultures described in (**e**). **e**, **f** *n* = 3 biological independent experiments. **g** Dot plot graph of the top 10 enriched KEGG pathways of DEGs from each comparison in human primary chondrocytes. **g** *n* = 4 replicates. **h** Schematic diagram representing molecular mechanisms modulating inflammation and ECM maintenance via LATS1-ZBTB20-PTEN axis

The results indicating that both TRULI and DAPA can restore ECM balance in cultured chondrocytes imply their potential therapeutic efficacy in attenuating OA progression. As a result, we conducted DMM surgery followed by drugs administration to assess their protective abilities. Based on our observations, the initial two weeks post-DMM surgery are considered a critical phase for the pathogenesis of OA, during which LATS1 undergoes phosphorylation, leading to the release of ZBTB20 for translocation into the nucleus. Therefore, we compared the impacts of TRULI and DAPA when given either immediately after the operation (TRULI_0 week, DAPA_0 week) or 2 weeks post-surgery (TRULI_2 weeks, DAPA_2 weeks) (Figs. 6a and S10A). A behavior test was performed using gait analysis to evaluate their therapeutic effects at 4/6/8 weeks post the surgery. As osteoarthritis develops, an increase in absolute paw angle, decrease in paw area, shorter stance time, and longer swing time were noted in the legs subjected to DMM surgery compared to the contralateral legs, indicative of OA-related pain. Administration of either TRULI or DAPA at both time points, immediately or 2 weeks post the surgery, improved the abnormal behaviors, particularly in the DAPA_0W group (Figs. 6b and S10B–D). Further exploration of the cartilage destruction in the defined mouse cohorts revealed that all administered medications demonstrated varying degrees of improvement in cartilage matrix loss and erosion at the cartilage periphery, among which TRULI_2 weeks and DAPA_0 week showed superior efficacy compared to the rest on restoring the thickness of hyaline cartilage (Fig. 6c, d). These results demonstrate that both TRULI and DAPA can alleviate OA progression, improving the abnormal behavior and moderating cartilage degeneration.

Next, we examined the beneficial effects of TRULI and DAPA in human cartilage that inhibiting matrix degradation ex vivo. The relative intact explants of the femoral head cartilage from patients receiving hip joint replacement were subjected to IL-1β, followed by supplement of TRULI and DAPA. The IL-1β induced surface erosion and loss of proteoglycan, as indicated by S.O. staining, were significantly and dose-dependently improved in cultures co-treated with TRULI or DAPA (Fig. 6e). Likewise, all groups treated with the compounds exhibited a noteworthy dose-dependent inhibitory influence on the release of glycosaminoglycans (GAGs), quantified via the dimethylmethylene blue (DMMB) assay (Fig. 6f). Summarily, these results showed the advantageous impacts of TRULI and DAPA on human cartilage.

Lastly, to gain a more profound comprehension of TRULI and DAPA mechanisms on ECM balance, we performed RNA-seq analysis using human primary cartilage chondrocytes, characterized by the expression of SOX9 and COL II (Fig. S11A), exposing to IL-1β solely or in conjunction with TRULI or DAPA. Integration of the top 10 enriched KEGG pathways from DEGs of each comparison reflects the feature changes of transcriptomes, indicating that both TRULI and DAPA treatment eliminated the association with various inflammation pathways including Measles, Kaposi sarcoma-associated herpesvirus infection, Influenza A and so on (Fig. 6g), further supporting our observations on ECM phenotypes. Nonetheless, various mechanisms indicated by the enriched KEGG pathways also account for the distinct effects of TRULI and DAPA on OA pathogenesis: TRULI predominantly influences the cell cycle, whereas DAPA primarily targets proteoglycans and ECM-receptor interactions (Figs. 6g and S11B–G). Collectively, these findings show that both TRULI and DAPA exhibit varying degrees of beneficial impacts in alleviating OA progress, attributed to the minor differences in the underlying mechanisms.

## Discussion

In this study, we demonstrate the contribution of the transcription factor ZBTB20 to inflammation response and ECM homeostasis in early-stage OA. ZBTB20 was observed to translocate into the nucleus at early-stage of OA, while gradually diminish as OA advanced. Chondrocytes-specific *Zbtb20*-deletion mice exhibited attenuated DMM-induced OA progress, recovered balance of ECM anabolism and catabolism. Mechanistically, the NF-κB signaling mediated disturbed ECM maintenance by ZBTB20 requires its suppression of *Pten* and consequent PI3K-Akt signaling activation. Furthermore, the subcellular localization as well as transcription activity of ZBTB20 was modulated in a LATS1 dependent manner. Attempts to modulate ZBTB20 by using TRULI and DAPA proved to restore ECM homeostasis, alleviate OA progression, improving the abnormal behavior and moderating cartilage degeneration.

Up to date, there is no approved cure for OA. Recently, strategies for OA treatment have transitioned from improving joint pain to preventing OA sequelae by early intervention prior to extensive damage, which requires comprehensive observations and definition of different stages of OA, particularly the initial phase.^24^ Nevertheless, once OA is diagnosed through clinical radiography, the disease has progressed significantly, may be resistant and irreversible to the current treatment. Furthermore, the attempts to define the early-stage knee OA clinically has turned out to be inconsistent that most studies included Kellgren-Lawrence (KL) grade of 2 or higher, reflecting advanced or later-stage of OA, hardly distinguishes the early-stage from later-stage OA.^25^ Taking advantage of animal models of experimental induced OA will improve the observation of disease progression and broaden the understanding of the underlying mechanisms, surpassing the limitations of defining the initial phase of osteoarthritis clinically. In this present study, we carried out DMM surgery in mice and continuously monitored the progress of OA at various time points. Interestingly, before the loss of cartilage ECM was detected, noticeable changes in chondrocyte morphology were observed, with chondrocytes appearing smaller at 2 weeks post-surgery. Given the decreases in PCM micromechanics as early as 3 days after surgery,^26^ the morphology change may be because of altered PCM properties, while molecular-level changes remain unexplored. In this study, we described the dynamic change of ZBTB20’s expression in cartilage chondrocytes during OA development that accumulation in the nucleus at 2 weeks post DMM surgery and gradually diminishing as OA advanced, and demonstrated the promoting role of ZBTB20 in OA development via triggering NF-κB signaling. Our results have defined a key protein, ZBTB20, in reflecting altered chondrocyte states during OA pathogenesis for the first time. In this context, these findings will enhance comprehension of the initiation and progression of osteoarthritis. Additionally, according to our founding, investigation on strategies based on ZBTB20 modulation in the initial phase of OA emerged to provide an effective anti-OA therapeutic approach.

In recent years, involvement of both local and systemic inflammation has been proved to be included in risk factors of OA, modulating the catabolism and anabolism in the joint.^6^ Our findings have demonstrated that ZBTB20 regulates the inflammatory response in chondrocytes through regulating NF-κB signaling, consistent to the previous studies of ZBTB20 triggering activity of NF-κB signaling in various processes including stress-induced visceral hypersensitivity, atherosclerosis, and cell migration and invasion of gastric cancer via indicated mechanisms.^27–30^ The potential significance of ZBTB20 in OA was highlighted due to the importance of the inflammatory processes in OA initiation and development and ZBTB20’s function in the inflammation response. Transcription factors are widely recognized for their essential function in signaling pathways, as they possess the capability to simultaneously modulate the expression of numerous genes.^31^ Our research has elucidated a novel mechanism by which ZBTB20 facilitates the activation of NF-κB signaling during OA progress, necessitating its inhibition of *Pten* and the subsequent activation of the PI3K-Akt pathway. Consequently, the inhibition of target gene expression, such as *Pten* and *IκBα*, by ZBTB20 establishes it as a vital modulator of the NF-κB signaling cascade.

Additionally, our founding uncovered a novel mechanism that the NF-κB signaling activated by ZBTB20 requires its suppression of *Pten* and consequent PI3K-Akt signaling activation. Remarkably, as reported in,^32^ the p-AKT level in cartilage increased significantly and rapidly in DMM-induced OA mice, accompanied with the founding that disruptive PTEN/Akt signaling initiates OA development. Thus, according to current research, approaches suppressing ZBTB20 activity or expression will rescue the disturbed PTEN/PI3K-Akt signaling and alleviate OA symptoms, providing a promising lead for osteoarthritis treatment. Intriguingly, study in hepatocytes revealed the essential role of PI3K-Akt signaling in the transcriptional activation process of the *iNOS* gene induced by IL-1β,^33^ highlighting the importance of targeting the PI3K-AKT signaling for intervening in the inflammation response. Additionally, several studies in cancer have suggested that PI3K-Akt signaling improves the transcriptional activity of NF-κB TFs, promoting and supporting the growth of tumors, as well as contributing to the development of multidrug-resistance.^34^ Hence the intervention of PI3K-Akt signaling by controlling ZBTB20 represents a promising therapeutic strategy for cancer treatment.

As a ubiquitous expressed transcription factor, ZBTB20 plays essential roles in organ development and growth, which has been reported to repress the expression of *Afp* and *ChREBP-α* in liver, *Fbp1* in pancreatic β cells, and regulate *Serca2α* in heart development.^7,35–37^ Additionally, the missense mutations in *ZBTB20*, residing within the *3q13.31* microdeletion syndrome critical region, underlie Primrose syndrome, a multisystem disorder disease.^38^ While the researchers have devoted more efforts on the downstream regulatory mechanisms, the question that how does the activity of ZBTB20 being modulated remains largely unknown. Interestingly, researchers has observed the nuclear translocation and increased transcription activity of ZBTB20 in hepatocytes upon high concentration of glucose treatment,^35^ suggesting the existence of possible regulatory mechanism. In the present work, we have detected the nucleus accumulation and activation of ZBTB20 in chondrocytes in early-stage OA, and identified the kinase LATS1 as the key factor controlling subcellular localization of ZBTB20. Our results demonstrated that the phosphorylation modification of LATS1 induced by IL-1β treatment abolishes the interaction of ZBTB20 and LATS1, therefore releases ZBTB20 to translocate into nucleus.

Regarding where the LATS1-ZBTB20 interaction takes place, LATS1 has been reported to exhibited different subcellular localizations, while mostly is activated in the cytoplasm in the classical Hippo signaling. Our findings also indicate that LATS1 primarily localizes within the cytoplasm of chondrocytes. On the other hand, we have observed the cytoplasm localization of ZBTB20 in chondrocytes via immunostaining and nucleus extraction assays. Previous research has discovered that ZBTB20 exhibits a cytoplasmic localization in hepatocytes cultured under conditions of low glucose.^35^ Given the avascular characteristics of articular cartilage, glucose permeates the cartilage via diffusion, leading to a hypoglycemic milieu within the chondrocytes.^39^ Studies indicate that even the deeper layers of cartilage may exhibit glucose concentrations as low as 1 mmol/L.^40^ Consequently, it is conceivable that the hypoglycemic conditions experienced by chondrocytes could play a role in the noted cytoplasmic alterations. Taken together, these results suggest that LATS1 interacts with ZBTB20 mostly in the cytoplasm.

According to our analysis of LATS1-dependent ZBTB20 cellular distribution rearrangement, we proposed that inhibition of LATS1 to hinder ZBTB20 function maybe provide a potential approach for OA treatment. The small molecule TRULI has been identified to block LATS1/2 activity and elicit proliferation of several cell types, while its effects on OA remains unclear.^41^ In the present study, administration of TRULI exhibited recovered ECM balance, slighter cartilage degeneration and improved behavior abnormality. However, the somewhat inadequate stability of TRULI restricts its in vivo application, necessitating frequent injections into the knee joint, which may lead to unforeseen cartilage damage. We noticed a slightly superior effectiveness of administering TRULI 2 weeks post-surgery compared to immediately after-surgery. The bio-material investigation into developing a sustained-release system will enhance the utilization of TRULI, requiring further exploration.

Besides the alteration in cellular distribution, we also observed a progressive decline in the expression of ZBTB20 as OA progressed and under IL-1β stimulation. According to the previous research, the expression of *Zbtb20* is under tightly control of microRNAs including miR-378a and miR-122.^42,43^ Interestingly, miR-378a is found to be most detectable in majority of late-stage OA synovial fluid and showed increased expression in IL-1β-stimulated OA synovial explants.^44^ Likewise, elevated expression of miR-122 was detected in OA cartilage compared to the health control tissue.^45^ Therefore, the upregulation of miR-378a and miR-122 may provide the explanation for the downregulation of *Zbtb20* in OA, the exact mechanism of which warrants further investigation. In addition to the potential mechanisms by which microRNAs such as miR-378a and miR-122 may down-regulate *Zbtb20* expression, the noted reduction in ZBTB20 levels could also be attributed to regulatory processes affecting the stability of the ZBTB20 protein. It has been reported that the BTB/POZ domain protein are known to interact with Cullin-ring ubiquitin ligases (CRLs), undergoing ubiquitination and subsequent degradation via the ubiquitin-proteasome system (UPS).^46^ Interestingly, our results suggest that overexpression of LATS1 induces increased protein level of ZBTB20 (Fig. S9E). Furthermore, supplement of TRULI restores the expression of ZBTB20 in IL-1β treated chondrocytes (Fig. S9F). Based on these findings, it is possible that LATS1-ZBTB20 interaction modulates the interaction between ZBTB20 and CRLs, hence preventing ZBTB20 ubiquitination and degradation by UPS.

On the other hand, we also tried to minimize ZBTB20 expression using dapagliflozin (DAPA), a small molecular compound which has been reported to suppress *Zbtb20* expression in renal tissues.^23^ Dapagliflozin is a prescription medication that is mainly used to help treat type 2 diabetes and heart failure.^47^ As a sodium-glucose transporter 2 (SGLT2) inhibitor, DAPA diminishes glucose reabsorption, enhances glucose excretion, and mitigates hyperglycemia.^47^ Several studies have shown the ability of DAPA to activate downstream SIRT1 signaling, attenuating endothelial-mesenchymal transition in myocardial fibrosis, ameliorating myocardial hypertrophy, thereby preventing cardiovascular damage.^48,49^ Additionally, DAPA has been shown to inhibit the PERK-eIF2α-CHOP pathway involved in the endoplasmic reticulum stress response by activating SIRT1, thereby mitigating endoplasmic reticulum stress-induced apoptosis in chondrocytes, which may help in preventing the progression of osteoarthritis.^50^ In our study, the supplement or oral taken of DAPA reduced the expression of ZBTB20, dramatically rescued the imbalance of ECM anabolism and catabolism, minimized the NF-κB signaling, improved the abnormal behavior, and alleviated OA progression. In addition, we observed that the sooner DAPA is taken, the more effective it is in treating OA. With the increasing appreciation, OA and diabetes often coexist due to their high prevalence and common risk factors.^51^ Given the fact that DAPA is a novel class of glucose-lowering agent and has been used to treat patients with type 2 diabetes clinically, our discovery of DAPA’s anti-OA therapeutic properties indicates a broader potential use of DAPA in treating both diabetes and osteoarthritis simultaneously.

## Materials and methods

### Ethical approval information, institution(s) and number(s)

All animal work performed in this study was approved by the Ethics in Animal Research Committee of the Air Force Medical University (20241307). Human articular cartilages in this study were collected from OA patients undergoing total knee/hip replacement surgery with the approval of the Medical Ethics Committee of the First Affiliated Hospital of the Air Force Medical University (KY20242189-C-1).

### Primary chondrocyte isolation and culture, transfection, immunoprecipitation, Western blot, and quantitative real time PCR (qPCR) analysis

To isolated primary chondrocytes, rib cartilage was dissected from P0-P3 *wildtype* or *Zbtb20*^*f/f*^ pups and digested with collagenase D (Roche) solution as described previously.^52^ The purified primary chondrocytes were resuspended with low-glucose DMEM (Gibco) supplemented with 10% fetal bovine serum (Gibco), 50 U/mL penicillin and 0.05 mg/mL streptomycin as a monolayer under sterile conditions at 37 °C under 5% CO_2_. Only the first passage cells were used for experiments.

The chondrocytes were treated with 1 ng/mL recombinant murine IL-1β (211-11B; PEPROTECH) for 48 h. For the treatment of the drugs, TRULI (HY-138489, MCE), and DAPA (short for Dapagliflozin, HY-10450, MCE) were supplemented with IL-1β to the primary chondrocytes.

To manipulate the expression of *Zbtb20* in chondrocytes, adenovirus expressing Cre recombinase (*Ad-Cre-GFP*) or *Zbtb20* (*Ad-mZbtb20-GFP*), and control adenovirus (*Ad-GFP*) (HANBIO) as control were transduced into *Zbtb20*^*f/f*^ or *wildtype* primary chondrocytes, followed by stimulation of IL-1β. Duplex siRNA targeting *Zbtb20* (GenePharma) were transfected into cells using Lipofectamine RNAiMAX (Invitrogen) in accordance with the manufacturer’s instructions, followed by treatment of IL-1β. The sequences of siRNAs used in this study are summarized in Table S1.

Immunoprecipitation, Western blot was performed according to standard protocols as previously described.^21^ The antibodies used in this study are summarized in Table S2. qPCR was performed as previously described.^53^ Mouse *Gapdh* was used as a normalized control. Primer sets are listed in Table S3.

The human primary chondrocytes were purchased from Procell (CP-H096), and cultured in medium specific for primary chondrocytes (CM-H096). The chondrocytes treated with 1 ng/mL recombinant human IL-1β (200-01B, PEPROTECH) together with TRULI or DAPA were subsequently subjected to RNA-seq analysis.

### RNA-seq、ATAC-seq、CUT&Tag-seq and corresponding analyses

#### RNA-seq

The *wildtype* primary chondrocytes treated by 1 ng/mL IL-1β for 0/6/48 h were harvested and lysed in TRIzol reagent (Invitrogen). The purified mRNA was quantified and qualified using the RNA Nano 6000 Assay Kit of the Bioanalyzer 2100 system (Agilent Technologies, CA, USA), and subjected to cDNA library preparation using NEBNext® Ultra™ RNA Library Prep Kit for Illumina®. The cDNA library was then sequenced by the Illumina NovaSeq 6000. Paired-end 150-bp clean reads were aligned to the *Mus Musculus* (GRCm38/mm10) reference genome using Hisat2 (v2.0.5). The differentially expressed genes (DEGs) were identified using the DESeq2 R package (1.20.0) with padj < 0.05. The union of all the DEGs from 3 groups of comparisons (6 h vs 0 h, 48 h vs 0 h, 48 h vs 6 h) emerged into a collection of 3 979 DEGs. These DEGs were clustered by the Mfuzz R package according to the expression profiles. Gene Ontology (GO) enrichment analysis of DEGs from each cluster was implemented by the clusterProfiler R package (3.8.1). The heatmap was plotted by SRplot, a free online platform for data visualization and graphing.^54^

The primary chondrocytes from *Zbtb20*^*f/f*^ pups transduced with *Ad-Cre-GFP* or *Ad-GFP* and treated with IL-1β were subjected to RNA-seq analysis as mentioned above. Multi-group difference scatter plot of all the DEGs from indicated groups of comparisons were plotted on OmicShare online platform. KEGG (http://www.genome.jp/kegg/) enrichment analysis of DEGs from indicated groups of comparisons were employed, and the top 10 KEGG pathways ranked by *P*-value of every comparison were plotted by SRplot.

The human primary chondrocytes treated with IL-1β and TRULI or DAPA were subjected to RNA-seq analysis as mentioned above. KEGG (http://www.genome.jp/kegg/) enrichment analysis of DEGs from indicated groups of comparisons were carried out. The top 10 KEGG pathways ranked by p-value of every comparison and the double volcano of the DEGs were plotted by SRplot.

#### ATAC-seq

ATAC-seq was performed as previously described.^55^ In brief, cells from each group were harvested, and lysed in lysis buffer. The Nextera DNA Library Preparation Kit (Illumina) was used to perform the transposition by incubating the nuclei with Nextera Tn5 Transposase at 37 °C for 30 min according to the manufacturer’s manual. The transposed DNA fragments were purified (MinElute PCR Purification Kit, Qiagen), and PCR-amplified using 1X NEBNext High-Fidelity PCR Master Mix (New England Biolabs, MA). Subsequently, the libraries were purified using the MinElute PCR Purification Kit (Qiagen) and subjected to sequencing on Illumina Novaseq 6000 using PE150. The clean reads were aligned to the reference genome sequences using the bwa program. ATAC peaks were called using MACS2 with cutoff *Q* value < 0.05 for each sample, and the overlapped peaks in samples from the same group were used for downstream analysis. Integrative Genomics Viewer (IGV) was used for the visualization of the ATAC peaks of *Col2α1*, *Acan*, *Cxcl1*, *Cxcl2*, *Cxcl3* in different samples. The specific peaks of each sample were identified using the DESeq2 R package. The HOMER’s findMotifsGenome.pl tool was then used for Motif analysis of these specific peaks.

#### CUT&Tag-seq

CUT&Tag-seq was performed as previously described.^19^ The *wildtype* primary chondrocytes treated by IL-1β or not were harvested, washed twice gently with wash buffer (20 mmol/L HEPES pH 7.5; 150 mmol/L NaCl; 0.5 mmol/L Spermidine; 1× Protease inhibitor cocktail), and incubated with Concanavalin A coated magnetic beads (Bangs Laboratories) at room temperature for 10 min. The beads-loaded cells were rinsed with dig wash buffer (20 mmol/L HEPES pH 7.5; 150 mmol/L NaCl; 0.5 mmol/L Spermidine; 1× Protease inhibitor cocktail; 0.05% Digitonin; 2 mmol/L EDTA) and then incubated with primary antibody (ZBTB20 Polyclonal antibody, 23987-1-AP, Proteintech) or normal rabbit IgG antibody (12-370, Millipore) on a rotating platform overnight at 4°C. After removing the primary antibody, the cells were incubated with secondary antibody (Anti-Rabbit IgG antibody, AP132, Millipore), and then pA-Tn5 adapter transposome complex, each step for 60 min at room temperature. Subsequently, DNA was purified using phenol-chloroform-isoamyl alcohol extraction and ethanol precipitation, PCR-amplified using 1X NEBNext High-Fidelity PCR Master Mix (New England Biolabs, MA), subjected to sequencing on Illumina Novaseq 6000 using 150 bp paired-end following the manufacturer’s instructions. The clean reads were aligned to the reference genome sequences with Bowtie2. IGV was used for the visualization of the peaks of ZBTB20 on *Pten* locus in different samples.

The seq data has been deposited under the GEO accession number (GSE269579, GSE269735).

#### Animals

All animal work performed in this study was approved by the Ethics in Animal Research Committee of the Air Force Medical University (20241307). In accordance with the standards for animal housing, mice were group housed at 23°C to 25°C with a 12-h light/dark cycle and allowed free access to water and standard laboratory pellets. Mice were raised under identical conditions according to the standards for animal housing.

*Zbtb20*^*f/f*^ mice^7^ were generously provided by Prof. Weiping Zhang, and mated with transgenic mice *Col2α1-CreER*^*T2*^^56^ to generate inducible chondrocyte-specific *Zbtb20* knockout (*Zbtb20*-icKO) mice. For the induction of knockout, 10-week-old *Zbtb20*^*f/f*^*; Col2α1-CreER*^*T2*^ mice and *Zbtb20*^*f/f*^ mice were intraperitoneally injected with Tamoxifen daily (1 mg/day for 5 consecutive days). Primer sets for genotyping are listed in Table S4.

To induce OA, the destabilization of the medial meniscus (DMM) surgery was carried out to *wildtype*, and *Zbtb20*-icKO male mice along with corresponding control mice at indicated age in the right knees as described previously.^57^ In sham surgery, the joint capsule was opened in the same fashion but without any further damage. The *wildtype* mice were euthanized at 2/4/8 weeks after surgery to monitor the changes of cartilage chondrocyte with OA progress. The *Zbtb20*-icKO mice were euthanized at 8/12 weeks after surgery to observe the degeneration of articular cartilage of indicated mice. Knee joints were collected for histological analyses and immunostaining analyses.

Therapy with drugs were initiated on the day of surgery or 2 weeks after surgery and continued to 8 weeks. The LATS1/2 inhibitor TRULI was dissolved by sonication into 10% Kolliphor HS 15 (42966, Sigma) in PBS solution (Gibco) at a concentration of 5 mg/mL. Mice undergoing DMM surgery then received intra-articular injection of 10 μL TRULI solution or the equivalent volume of vehicle (10% Kolliphor HS 15 in PBS) twice a week as shown in Fig. 6a. DAPA was dissolved in H_2_O by sonication at a concentration of 5 mg/mL and assessed in DMM mice at single dose of 5 mg per kg of body weight by oral gavage every day as shown in Fig. 6a. The body weight and blood glucose of indicated group of mice were monitored during the administration of drugs. The mice were subjected to gait analyses at 4/6/8 weeks after surgery, and euthanized by the end of the therapy to compare the degeneration of articular cartilage in different groups.

### Collection and preparation of human articular cartilage explants

Human articular cartilages were collected from OA patients undergoing total knee replacement surgery with the approval of the Medical Ethics Committee of the First Affiliated Hospital of the Air Force Medical University (KY20242189-C-1). To compare the difference between damaged and undamaged cartilage and avoid the variation among patients, the cartilages were harvested from the lateral (undamaged) and medial (damaged) region of the tibia plateaus (as shown in Fig. 1).

The whole height cartilage explants were subjected to histology analyses and IHC analyses.

The cartilage tissues were cut into pieces and lysed in RIPA buffer supplemented with protease inhibitor (P8340, Sigma). WB was employed to analyze the expression pattern of ZBTB20 and COL II between the undamaged and damaged region.

The cartilage explants for DMMB assay and S.O. staining was harvested, cut into pieces (3 mm × 3 mm) and cultured in DMEM low-glucose medium (Gibco) supplemented with 10% fetal bovine serum (Gibco), 50 U/mL penicillin and 0.05 mg/mL streptomycin for 24 h. The medium was then replaced by medium supplemented with IL-1β (P10749; PEPROTECH) in the presence or absence of TURLI (HY-138489, MCE), DAPA(HY-10450, MCE) for 5 days.

### Histology analysis

Mouse joints were fixed with 4% paraformaldehyde overnight, decalcified with 10% EDTA for 7 days. The full thickness cartilage tissues from patients were fixed by 4% paraformaldehyde overnight, decalcified with 10% EDTA for 12 weeks. The cultured human cartilage explants were fixed by 4% paraformaldehyde overnight, decalcified with 10% EDTA for 24 h. The samples were embedded in O.C.T. compound, and sectioned using a frozen slicer. Safranin O/Fast Green staining was employed to analyze phenotypic changes within the cartilage tissues. Images were captured with an Olympus microscope. The articular cartilage destruction was quantified using the established Osteoarthritis Research Society International (OARSI) scoring system (score, 0–6).^15^

### Immunostaining

Immunostaining was performed according to the standard protocol. The antibodies used in this study are summarized in Table S2.

Immunohistochemistry (IHC) staining was performed on PFA-fixed tissue frozen sections accordingly as described before.^53^ Images were acquired with an Olympus microscope.

For immunofluorescence analyses of the cartilage and the primary chondrocytes, images were captured with a laser-scanning confocal microscope (Zeiss LSM 900) using the ZEN 2 (Zeiss), and analyzed using ImageJ (1.53f51) software.

### ChIP-qPCR

ChIP assays were performed in primary chondrocytes transduced with *Ad-Flag-Zbtb20* using the Pierce Agarose ChIP Kit (26156, Thermo) in accordance with the manufacturer’s instructions. qPCR was performed according to standard protocols as previously described to quantify the enrichment of DNA. The sequences of primers used in this study are summarized in Table S3.

### Single cell RNA-seq data analysis

ScRNA-Seq data and cell annotation tables were obtained from the GEO datasets (GSE104782). The quality-passed cells were used for downstream analysis. We normalized the data using the R package Seurat (version 5.0.). Then we performed principal component analysis (PCA) using the highly variable genes (Top 2 000), built a Shared Nearest Neighbor (SNN) Graph using the top 30. We next applied the Louvain algorithm to cluster the cells (top 30 PCs), with the resolution parameter of 0.5. The expression of the gene set of ZBTB20 target genes in indicated cell clusters was evaluated using the R package AUCell.

### Proximity labeling with TurboID and TurboID-ZBTB20

The proximity labeling assays in chondrocytes transduced *Ad-TurboID-GFP* or *Ad-TurboID-Fg-Zbtb20* were performed as previously described.^58^ In brief, 48 h after adenovirus infection, the cells were incubated with warm biotin-containing (50 μmol/L) medium for 15 min at 37°C for labeling, and then rinsed by ice-cold PBS to stop the labeling reaction. The cells were harvested, lysed in RIPA lysis buffer supplemented with protease inhibitor. The streptavidin magnetic beads were added to the labeled cell lysates to enrich the biotin-labeled proteins by rotating at 4°C overnight. The beads were then washed twice with RIPA lysis buffer, once with 1 mol/L KCl, once with 0.1 mol/L Na_2_CO_3_, once with 2 mol/L urea in 10 mmol/L Tris-HCl (pH 8.0), and twice with RIPA lysis buffer. The protein samples were eluted from the beads using protein loading buffer supplemented with 2 mmol/L biotin and 20 mmol/L DTT at 95°C for 5 min, and analyzed by western blot.

### DMMB assay

DMMB assay was employed to quantify GAGs secreted by the cultured human cartilage explants to the medium. The indicated medium was mixed with DMMB reagent, and the absorbance at 525 nm was measured using a microplate reader (Synergy H1M, BioTek). The release of GAGs was calculated by normalizing the amounts of secreted GAGs to the weight of the cartilage explants.

### Gait analysis

Automated gait analysis was performed on walking mice using the MSI DigiGait^TM^ Imaging System as previously reported,^59^ by imaging the animals from below a transparent treadmill. The software quantified the characteristics of gait, including step sequence patterns, stride length, cadence, and paw placement. The automated gait analysis of mice from every group were conducted at 4/6/8 weeks after DMM surgery. Comparison was made between the right hind paw and left hind paw in each run of each animal. We calculated the following parameters: RH/LH absolute paw angle, RH/LH paw area, RH/LH stance time, RH/LH swing time.

### Statistical analysis

Statistical analysis was performed with GraphPad Prism version 9.3.0, and the results were given as mean ± SEM. Differences between experimental groups were assessed using one-way ANOVA with Sidak post hoc test or two-way ANOVA with Turkey HSD post hoc test: * is *P* < 0.05, ** is *P* < 0.01, *** is *P* < 0.001, ns is no significance with *P* > 0.05.

## Supplementary information

Supplementary materials

## Data Availability

Data are available in a public, open access repository. All data supporting the findings of this study are available within the article or from the corresponding authors upon reasonable request.

## References

[1] Hunter, D. J. & Bierma-Zeinstra, S. Osteoarthritis. *Lancet* **393**, 1745–1759 (2019). 31034380 10.1016/S0140-6736(19)30417-9

[2] Leifer, V. P., Katz, J. N. & Losina, E. The burden of OA-health services and economics. *Osteoarthr. Cartil.* **30**, 10–16 (2022). 10.1016/j.joca.2021.05.007 PMC8605034 34023527

[3] Salmon, J. H. et al. Economic impact of lower-limb osteoarthritis worldwide: a systematic review of cost-of-illness studies. *Osteoarthr. Cartil.* **24**, 1500–1508 (2016). 10.1016/j.joca.2016.03.012 27034093

[4] Sharma, L. Osteoarthritis of the Knee. *N. Engl. J. Med.* **384**, 51–59 (2021). 33406330 10.1056/NEJMcp1903768

[5] Mahmoudian, A., Lohmander, L. S., Mobasheri, A., Englund, M. & Luyten, F. P. Early-stage symptomatic osteoarthritis of the knee - time for action. *Nat. Rev. Rheumatol.* **17**, 621–632 (2021). 34465902 10.1038/s41584-021-00673-4

[6] van den Bosch, M. H. J., Blom, A. B. & van der Kraan, P. M. Inflammation in osteoarthritis: Our view on its presence and involvement in disease development over the years. *Osteoarthr. Cartil.* **32**, 355–364 (2024). 10.1016/j.joca.2023.12.005 38142733

[7] Xie, Z. et al. Zinc finger protein ZBTB20 is a key repressor of alpha-fetoprotein gene transcription in liver. *Proc. Natl. Acad. Sci. USA* **105**, 10859–10864 (2008). 18669658 10.1073/pnas.0800647105 PMC2504784

[8] Li, H. et al. ZBTB20 regulates plasma triglyceride metabolism by repressing lipoprotein lipase gene transcription in hepatocytes. *Hepatology (Baltimore, Md.)*, **75**, 1169–1180 (2021). 10.1002/hep.32176 PMC9118135 34580885

[9] Xie, Z. et al. Zbtb20 is essential for the specification of CA1 field identity in the developing hippocampus. *Proc. Natl. Acad. Sci. USA* **107**, 6510–6515 (2010). 20308569 10.1073/pnas.0912315107 PMC2851958

[10] Zhou, G. et al. Zbtb20 regulates the terminal differentiation of hypertrophic chondrocytes via repression of Sox9. *Dev. (Camb., Engl.)* **142**, 385–393 (2015). 10.1242/dev.108530 25564625

[11] Jenei-Lanzl, Z., Meurer, A. & Zaucke, F. Interleukin-1β signaling in osteoarthritis - chondrocytes in focus. *Cell. Signal.* **53**, 212–223 (2019). 30312659 10.1016/j.cellsig.2018.10.005

[12] Barakat, S. et al. Dimerization choice and alternative functions of ZBTB transcription factors. *FEBS J.* **291**, 237–255 (2024). 37450366 10.1111/febs.16905

[13] Zhong, L. et al. Single cell transcriptomics identifies a unique adipose lineage cell population that regulates bone marrow environment. *ELife* **9**, e54695 (2020). 10.7554/eLife.54695 PMC7220380 32286228

[14] Bernabei, I., So, A., Busso, N. & Nasi, S. Cartilage calcification in osteoarthritis: mechanisms and clinical relevance. *Nat. Rev. Rheumatol.* **19**, 10–27 (2023). 36509917 10.1038/s41584-022-00875-4

[15] Pritzker, K. P. H. et al. Osteoarthritis cartilage histopathology: grading and staging. *Osteoarthr. Cartil.* **14**, 13–29 (2006). 10.1016/j.joca.2005.07.014 16242352

[16] Burr, D. B. & Gallant, M. A. Bone remodelling in osteoarthritis. *Nat. Rev. Rheumatol.* **8**, 665–673 (2012). 22868925 10.1038/nrrheum.2012.130

[17] van der Kraan, P. M. & van den Berg, W. B. Chondrocyte hypertrophy and osteoarthritis: role in initiation and progression of cartilage degeneration? *Osteoarthr. Cartil.* **20**, 223–232 (2012). 10.1016/j.joca.2011.12.003 22178514

[18] Peng, Z. et al. The regulation of cartilage extracellular matrix homeostasis in joint cartilage degeneration and regeneration. *Biomaterials* **268**, 120555 (2021). 33285440 10.1016/j.biomaterials.2020.120555

[19] Kaya-Okur, H. S. et al. CUT&Tag for efficient epigenomic profiling of small samples and single cells. *Nat. Commun.* **10**, 1930 (2019). 31036827 10.1038/s41467-019-09982-5 PMC6488672

[20] Ji, Q. et al. Single-cell RNA-seq analysis reveals the progression of human osteoarthritis. *Ann. Rheum. Dis.* **78**, 100–110 (2019). 30026257 10.1136/annrheumdis-2017-212863 PMC6317448

[21] Hao, X. et al. Lola regulates Drosophila adult midgut homeostasis via non-canonical hippo signaling. *Elife* **9**, e47542 (2020). 10.7554/eLife.47542 PMC7299341 31934851

[22] Ma, S., Meng, Z., Chen, R. & Guan, K.-L. The Hippo Pathway: Biology and Pathophysiology. *Annu. Rev. Biochem.* **88**, 577–604 (2019). 30566373 10.1146/annurev-biochem-013118-111829

[23] Wei, J. et al. RNA-Seq transcriptome analysis of renal tissue from spontaneously hypertensive rats revealed renal protective effects of dapagliflozin, an inhibitor of sodium-glucose cotransporter 2. *Eur. J. Pharmaceutical Sci.: Offic. J. Eur. Federation Pharmaceutical Sci.* **189**, 106531 (2023). 10.1016/j.ejps.2023.106531 37479045

[24] Yao, Q. et al. Osteoarthritis: pathogenic signaling pathways and therapeutic targets. *Signal Transduct. Target Ther.* **8**, 56 (2023). 36737426 10.1038/s41392-023-01330-w PMC9898571

[25] Liew, J. W. et al. A scoping review of how early-stage knee osteoarthritis has been defined. *Osteoarthr. Cartil.* **31**, 1234–1241 (2023). 10.1016/j.joca.2023.04.015 PMC10528892 37225053

[26] Chery, D. R. et al. Early changes in cartilage pericellular matrix micromechanobiology portend the onset of post-traumatic osteoarthritis. *Acta Biomater.* **111**, 267–278 (2020). 32428685 10.1016/j.actbio.2020.05.005 PMC7321882

[27] Luo, Q.-Q. et al. ZBTB20 mediates stress-induced visceral hypersensitivity via activating the NF-κB/transient receptor potential channel pathway. *Neurogastroenterol. Motil.* **36**, e14718 (2024). 38009899 10.1111/nmo.14718

[28] Tao, J. et al. ZBTB20 positively regulates oxidative stress, mitochondrial fission, and inflammatory responses of ox-LDL-induced macrophages in atherosclerosis. *Oxid. Med. Cell. Longev.* **2021**, 5590855 (2021). 33777314 10.1155/2021/5590855 PMC7972849

[29] Qiu, J. et al. ZBTB20-mediated titanium particle-induced peri-implant osteolysis by promoting macrophage inflammatory responses. *Biomater. Sci.* **8**, 3147–3163 (2020). 32363359 10.1039/d0bm00147c

[30] Zhang, Y. et al. ZBTB20 promotes cell migration and invasion of gastric cancer by inhibiting IkappaBalpha to induce NF-kappaB activation. *Artif. Cells Nanomed. Biotechnol.* **47**, 3862–3872 (2019). 31556767 10.1080/21691401.2019.1670188

[31] Lambert, S. A. et al. The human transcription factors. *Cell* **172**, 650–665 (2018). 29425488 10.1016/j.cell.2018.01.029 PMC12908702

[32] Xie, J. et al. Sustained Akt signaling in articular chondrocytes causes osteoarthritis via oxidative stress-induced senescence in mice. *Bone Res.* **7**, 23 (2019). 31646013 10.1038/s41413-019-0062-y PMC6804644

[33] Teshima, S. et al. Up-regulation of IL-1 receptor through PI3K/Akt is essential for the induction of iNOS gene expression in hepatocytes. *J. Hepatol.* **40**, 616–623 (2004). 15030977 10.1016/j.jhep.2003.12.018

[34] Rascio, F. et al. The pathogenic role of PI3K/AKT pathway in cancer onset and drug resistance: an updated review. *Cancers* **16**, 3949 (2021). 10.3390/cancers13163949 PMC8394096 34439105

[35] Liu, G. et al. Regulation of hepatic lipogenesis by the zinc finger protein Zbtb20. *Nat. Commun.* **8**, 14824 (2017). 28327662 10.1038/ncomms14824 PMC5364431

[36] Zhang, Y. et al. The zinc finger protein ZBTB20 regulates transcription of fructose-1,6-bisphosphatase 1 and β cell function in mice. *Gastroenterology* **142**, 1571–1580.e1576 (2012). 22374165 10.1053/j.gastro.2012.02.043

[37] Ren, A.-J. et al. ZBTB20 regulates SERCA2a activity and myocardial contractility through phospholamban. *Circ. Res.* **134**, 252–265 (2024). 38166470 10.1161/CIRCRESAHA.123.323798

[38] Cordeddu, V. et al. Mutations in ZBTB20 cause Primrose syndrome. *Nat. Genet.* **46**, 815–817 (2014). 25017102 10.1038/ng.3035

[39] Zhou, S., Cui, Z. & Urban, J. P. G. Factors influencing the oxygen concentration gradient from the synovial surface of articular cartilage to the cartilage-bone interface: a modeling study. *Arthritis Rheumatism* **50**, 3915–3924 (2004). 15593204 10.1002/art.20675

[40] Heywood, H. K., Knight, M. M. & Lee, D. A. Both superficial and deep zone articular chondrocyte subpopulations exhibit the Crabtree effect but have different basal oxygen consumption rates. *J. Cell. Physiol.* **223**, 630–639 (2010). 20143333 10.1002/jcp.22061

[41] Kastan, N. R. et al. Development of an improved inhibitor of Lats kinases to promote regeneration of mammalian organs. *Proc. Natl. Acad. Sci. USA* **119**, e2206113119 (2022). 35867764 10.1073/pnas.2206113119 PMC9282237

[42] Wang, J., Liu, Z. H. & Yu, L. J. Long non-coding RNA LINC00641 promotes cell growth and migration through modulating miR-378a/ZBTB20 axis in acute myeloid leukemia. *Eur. Rev. Med. Pharm. Sci.* **23**, 7498–7509 (2019). 10.26355/eurrev_201909_18864 31539138

[43] Kojima, K. et al. MicroRNA122 is a key regulator of α-fetoprotein expression and influences the aggressiveness of hepatocellular carcinoma. *Nat. Commun.* **2**, 338 (2011). 21654638 10.1038/ncomms1345

[44] Li, Y. H. et al. Identification of synovial fluid microRNA signature in knee osteoarthritis: differentiating early- and late-stage knee osteoarthritis. *Osteoarthr. Cartil.* **24**, 1577–1586 (2016). 10.1016/j.joca.2016.04.019 27143365

[45] Bai, Y., Chen, K., Zhan, J. & Wu, M. miR-122/SIRT1 axis regulates chondrocyte extracellular matrix degradation in osteoarthritis. *Bioscience Reports* **40**, BSR20191908 (2020). 10.1042/BSR20191908 PMC7308613 32395770

[46] Choi, S.-H., Cho, S.-Y., Park, S. Y. & Hur, M.-W. Post-translational regulation of proto-oncogene ZBTB7A expression by p53 status in cancer cells: HSP90-dependent stabilization vs. p53-KLHL20-ubiquitin proteasomal degradation. *Biochimica Et. Biophysica Acta Gene Regul. Mech.* **1866**, 194931 (2023). 10.1016/j.bbagrm.2023.194931 37011832

[47] Dhillon, S. Dapagliflozin: a review in type 2 diabetes. *Drugs* **79**, 1135–1146 (2019). 31236801 10.1007/s40265-019-01148-3 PMC6879440

[48] Wang, W. et al. SIRT1 mediates the inhibitory effect of Dapagliflozin on EndMT by inhibiting the acetylation of endothelium Notch1. *Cardiovasc. Diabetol.* **22**, 331 (2023). 38017499 10.1186/s12933-023-02040-x PMC10685714

[49] Yang, J., Li, L., Zheng, X., Lu, Z. & Zhou, H. Dapagliflozin attenuates myocardial hypertrophy via activating the SIRT1/HIF-1α signaling pathway. *Biomed. Pharmacother. = Biomed. Pharmacotherapie* **165**, 115125 (2023). 10.1016/j.biopha.2023.115125 37421782

[50] Liu, Z. et al. Dapagliflozin suppress endoplasmic reticulum stress mediated apoptosis of chondrocytes by activating Sirt1. *Chem.-Biol. Interact.* **384**, 110724 (2023). 37741535 10.1016/j.cbi.2023.110724

[51] Wei, G. et al. Risk of metabolic abnormalities in osteoarthritis: a new perspective to understand its pathological mechanisms. *Bone Res.* **11**, 63 (2023). 38052778 10.1038/s41413-023-00301-9 PMC10698167

[52] Gosset, M., Berenbaum, F., Thirion, S. & Jacques, C. Primary culture and phenotyping of murine chondrocytes. *Nat. Protoc.* **3**, 1253–1260 (2008). 18714293 10.1038/nprot.2008.95

[53] Hao, X. et al. XMU-MP-1 attenuates osteoarthritis via inhibiting cartilage degradation and chondrocyte apoptosis. *Front. Bioeng. Biotechnol.* **10**, 998077 (2022). 36199358 10.3389/fbioe.2022.998077 PMC9527278

[54] Tang, D. et al. SRplot: A free online platform for data visualization and graphing. *PLoS One* **18**, e0294236 (2023). 37943830 10.1371/journal.pone.0294236 PMC10635526

[55] Buenrostro, J. D., Wu, B., Chang, H. Y. & Greenleaf, W. J. ATAC-seq: a method for assaying chromatin accessibility genome-wide. *Curr. Protoc. Mol. Biol.* **109**, 21.29.21–21.29.29 (2015). 10.1002/0471142727.mb2129s109 PMC4374986 25559105

[56] Chen, M. et al. Generation of a transgenic mouse model with chondrocyte-specific and tamoxifen-inducible expression of Cre recombinase. *Genes. (N. Y., N. Y. : 2000)* **45**, 44–50 (2007). 10.1002/dvg.20261 PMC2654410 17211877

[57] Glasson, S. S., Blanchet, T. J. & Morris, E. A. The surgical destabilization of the medial meniscus (DMM) model of osteoarthritis in the 129/SvEv mouse. *Osteoarthr. Cartil.* **15**, 1061–1069 (2007). 10.1016/j.joca.2007.03.006 17470400

[58] Cho, K. F. et al. Proximity labeling in mammalian cells with TurboID and split-TurboID. *Nat. Protoc.* **15**, 3971–3999 (2020). 33139955 10.1038/s41596-020-0399-0

[59] Dorman, C. W., Krug, H. E., Frizelle, S. P., Funkenbusch, S. & Mahowald, M. L. A comparison of DigiGait™ and TreadScan™ imaging systems: assessment of pain using gait analysis in murine monoarthritis. *J. Pain. Res.* **7**, 25–35 (2014). 24516338 10.2147/JPR.S52195 PMC3883276

