# Supplementary material for: LATS1-modulated ZBTB20 perturbing cartilage matrix homeostasis contributes to early-stage osteoarthritis
Source: Bone Res. 2025 Mar 12;13:33. doi: 10.1038/s41413-025-00414-3 (PMC11897192; doi:10.1038/s41413-025-00414-3)
Supplement: Supplementary file 1 — Supplementary materials [file 41413_2025_414_MOESM1_ESM.pdf]

LATS1-modulated ZBTB20 perturbing cartilage matrix homeostasis contributes to early-stage osteoarthritis

Xue Hao *et al.*

*Corresponding author. Email: jieqiangchina@126.com (Lead contact), yangliu@fmmu.edu.cn, wzhang@smmu.edu.cn

This PDF file includes:

Figs. S1 to S11
Tables S1 to S4

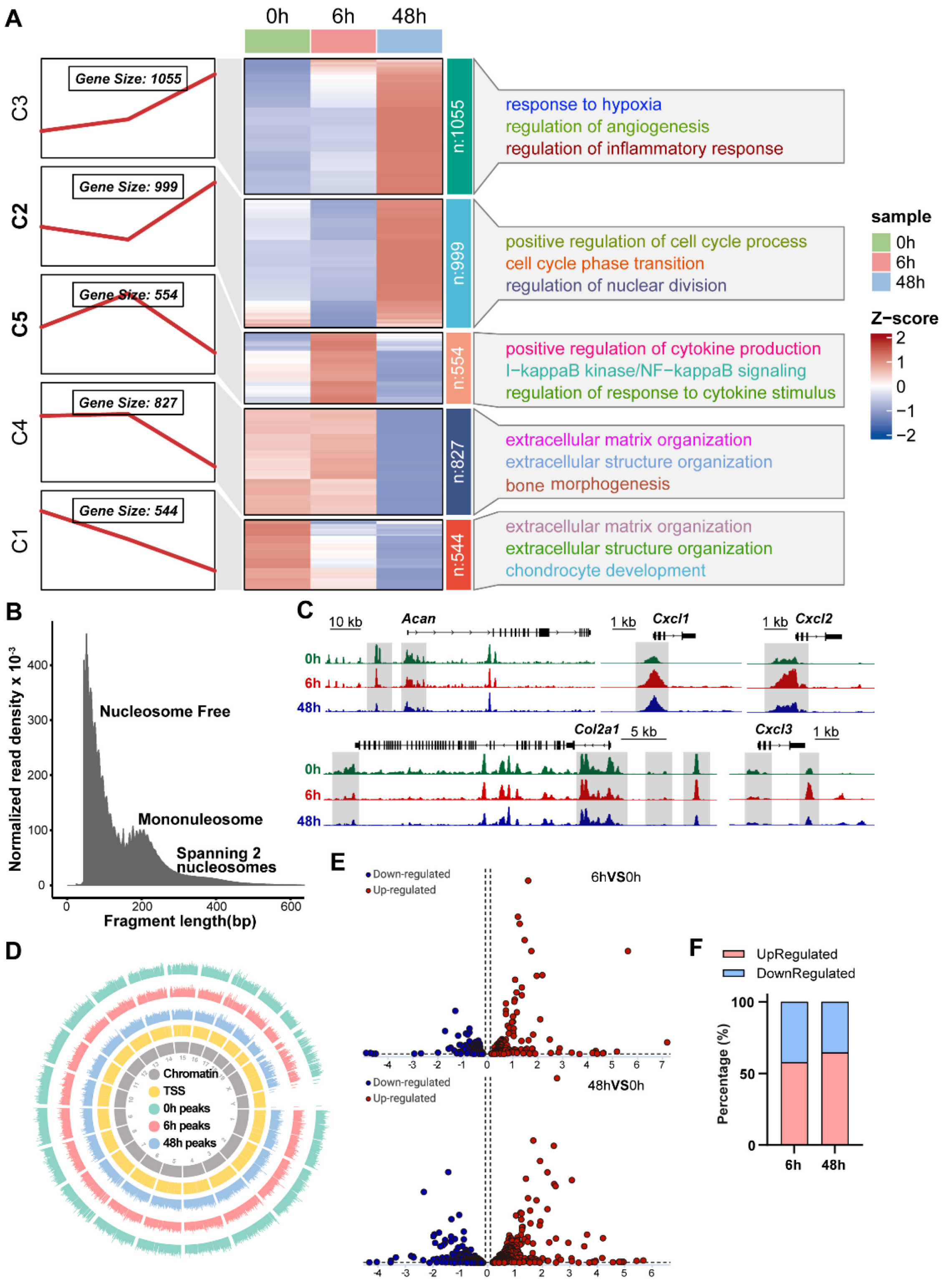

Fig. S1. ZBTB20 is an important transcription factor in early-OA (A) Heatmap showing the expression profiles of DEGs in chondrocytes treated for 0/6/48h, categorized into 5 clusters. Panels on the left is curves showing the expression trend of genes in the clusters. Panel on the

right is the GO analysis results of the indicated genes within the clusters. (**B**) Density of ATAC-seq fragments patterns of mono- and di-nucleosomes. (**C**) Representative genes with changed peaks of ATAC-seq reads in promoters. (**D**) Circos plot showing genome wide chromatin accessibility in chondrocytes treated by IL-1β for 0/6/48h. (**E**) Volcano plot of DEGs in each comparison. The top panel is DEGs of 6h versus 0h. The bottom panel is 48h versus 0h. (**F**) Graph showing the portion of upregulated and downregulated DEGs in each comparison. (**A**)-(**F**): n= 3 replicates.

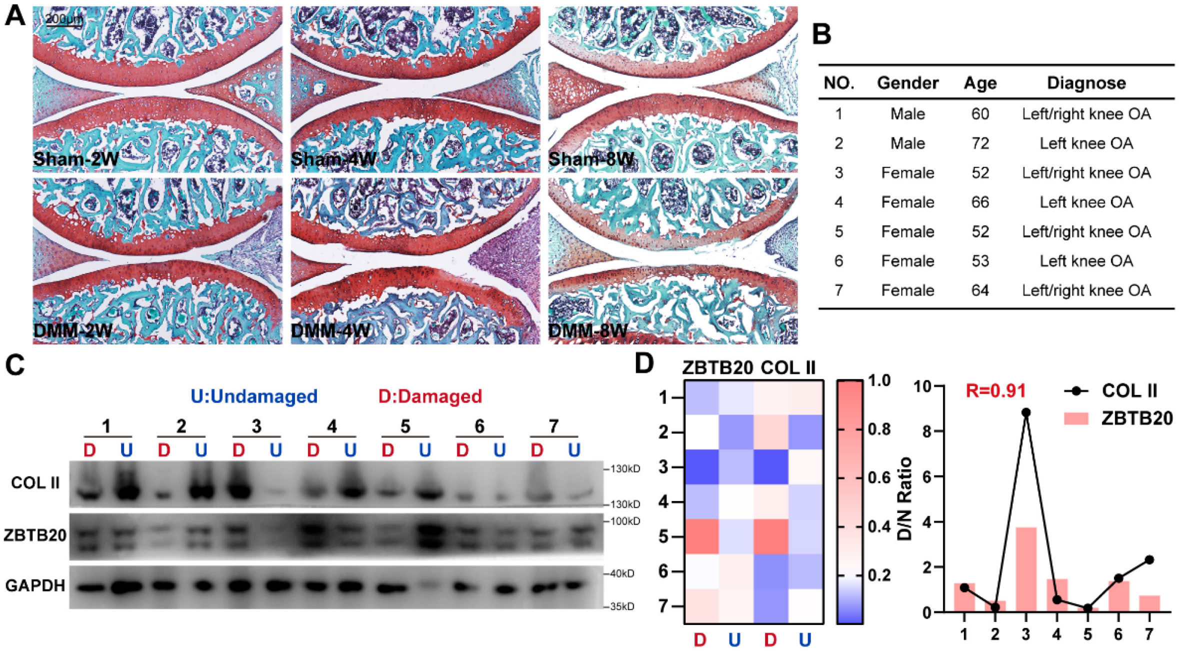

NO.	Gender	Age	Diagnose
1	Male	60	Left/right knee OA
2	Male	72	Left knee OA
3	Female	52	Left/right knee OA
4	Female	66	Left knee OA
5	Female	52	Left/right knee OA
6	Female	53	Left knee OA
7	Female	64	Left/right knee OA

Fig. S2. ZBTB20 is an important transcription factor in early-OA (**A**) Representative images of Safranin O/Fast Green staining of articular cartilage from mice underwent DMM or Sham surgery for 2/4/8W. n= 3 mice per group. (**B**) Information of OA patients providing tibial plateau cartilage tissue samples. (**C**) Western blot of COL II and ZBTB20 in the cartilage samples from the relative damaged and undamaged regions. (**D**) Statistical analysis of the expression of COL II and ZBTB20 in (**C**). Panel on the left is heatmap showing the expression of COL II and ZBTB20. Panel on the right is curves of COL II and ZBTB20 expressions.

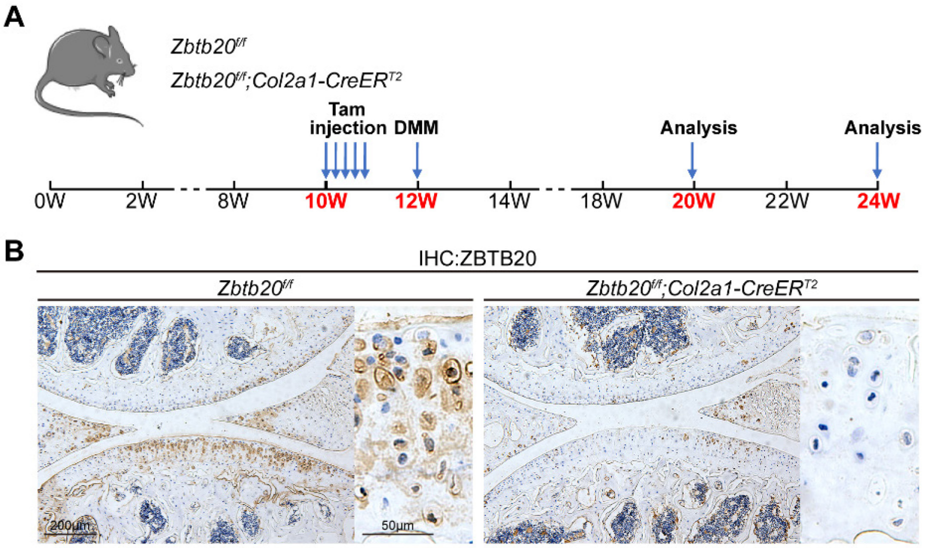

Fig. S3. Validation for knockout efficiency of *Zbtb20*-icKO mice. (**A**) Schematic diagram of TAM-induced *Zbtb20* knockout in chondrocytes, and following OA model by DMM surgery. (**B**) Representative images of IHC staining of ZBTB20 in articular cartilage from indicated mice. n= 3 mice per group.

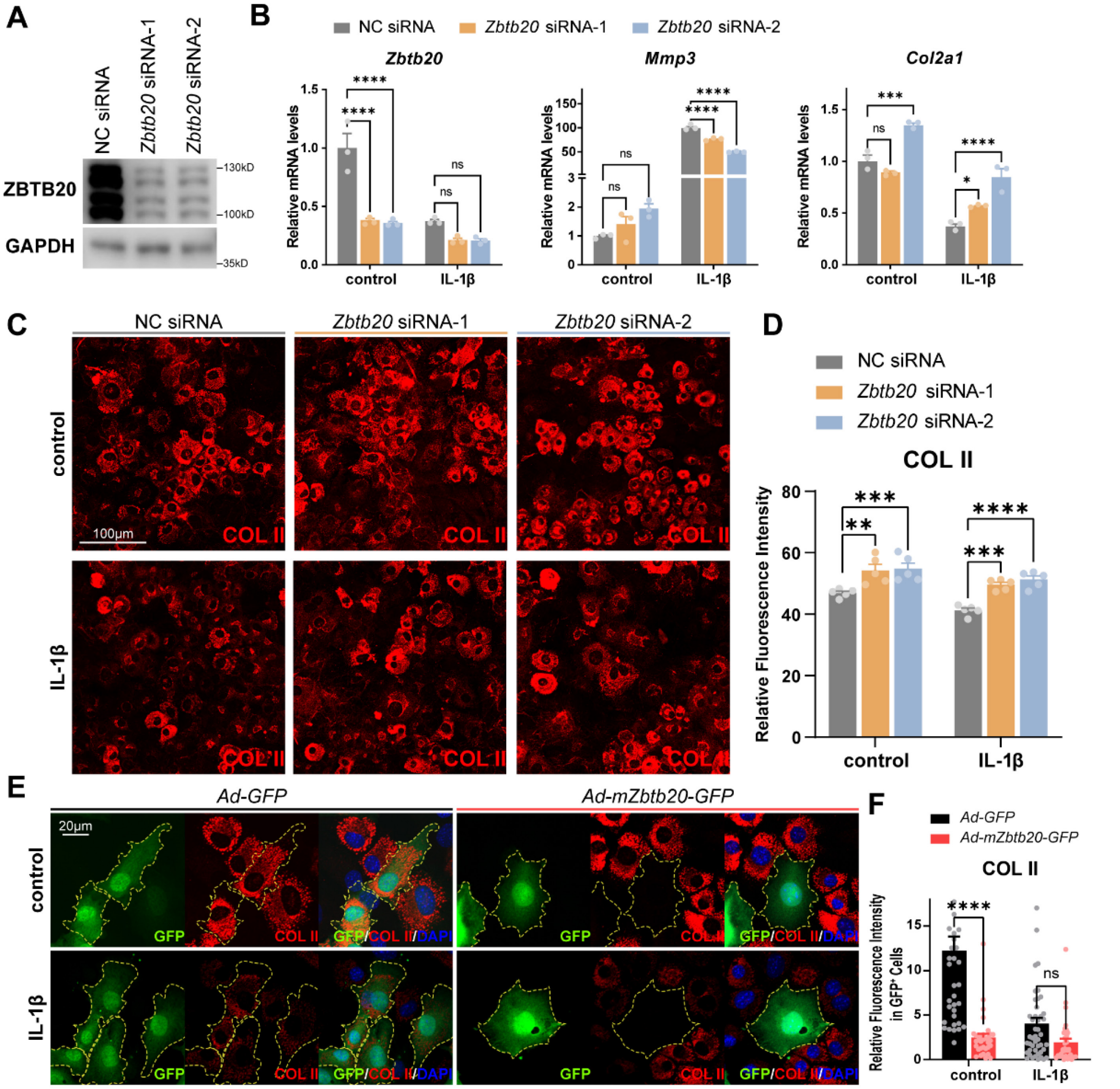

Fig. S4. *Zbtb20* knockout restores ECM homeostasis (**A**) Western blot of ZBTB20 in primary chondrocytes transfected with control or *Zbtb20* siRNAs. (**B**) Relative mRNA levels of *Zbtb20*, *Mmp3*, and *Col2α1* in chondrocytes transfected with control or *Zbtb20* siRNAs, treated with IL-1β or not. (**A**)-(**B**): n= 3 biological independent experiments. (**C**) Representative images of immunofluorescence staining of COL II in chondrocytes transfected with control or *Zbtb20* siRNA, treated with IL-1β or not. n= 3 biological independent experiments. (**D**) Statistical analysis of the relative fluorescence intensity of COL II in indicated groups of cells in (**C**). n= 5 views per group for one biological replicate. (**E**) Representative images of immunofluorescence staining of COL II in chondrocytes transfected with *Ad-GFP* or *Ad-mZbtb20-GFP*, treated with IL-1β or not. The dashed lines marked the GFP^+ cells. n= 3 biological independent experiments. (**F**) Statistical analysis of relative fluorescence intensity of COL II in GFP^+ chondrocytes of

indicated groups of cells in (**E**). n= 37, 33, 46, 34 GFP^+ cells quantified for one biological replicate.

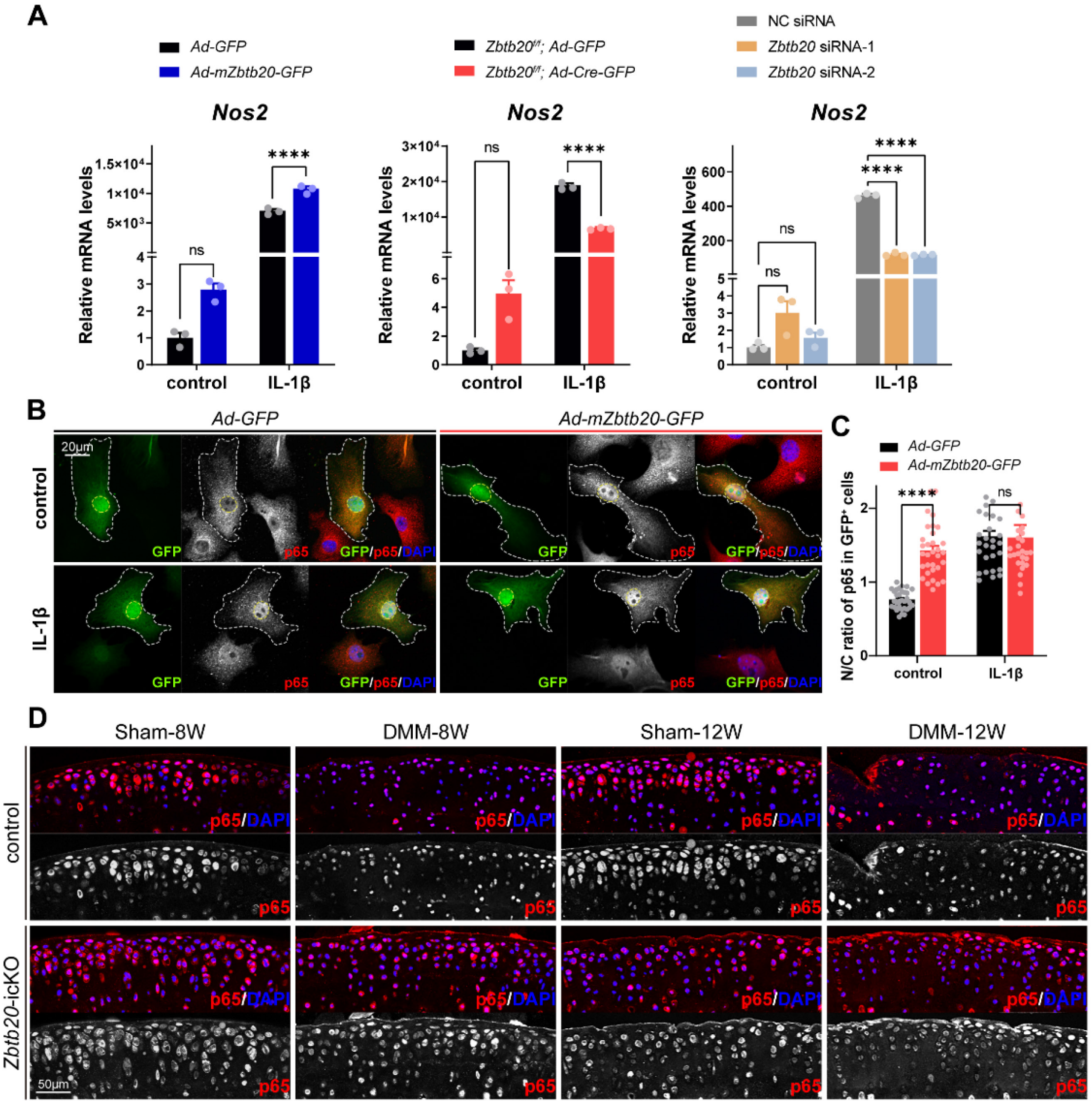

Fig. S5. ZBTB20 triggers NF-κB signaling (**A**) Relative mRNA levels of *Nos2* in *Zbtb20* overexpression or knockdown chondrocytes followed by IL-1B stimulation. n= 3 biological independent experiments. (**B**) Representative images of immunofluorescence staining of p65 in chondrocytes transduced with *Ad-GFP* or *Ad-mZbtb20-GFP*, treated with IL-1β or not. The dashed lines mark the GFP^+ chondrocytes. The dashed circles mark the nucleus. n= 3 biological independent experiments. (**C**) Statistical analysis of the N/C ratio of p65 in GFP^+ chondrocytes in indicated groups of cells in (**B**). n= 32, 32, 30, 26 GFP^+ cells quantified for one biological replicate. (**D**) Representative images of immunofluorescence staining of p65 in articular cartilage from *Zbtb20*-icKO or corresponding control mice underwent DMM or Sham surgery for 8/12W. n= 3 mice per group.

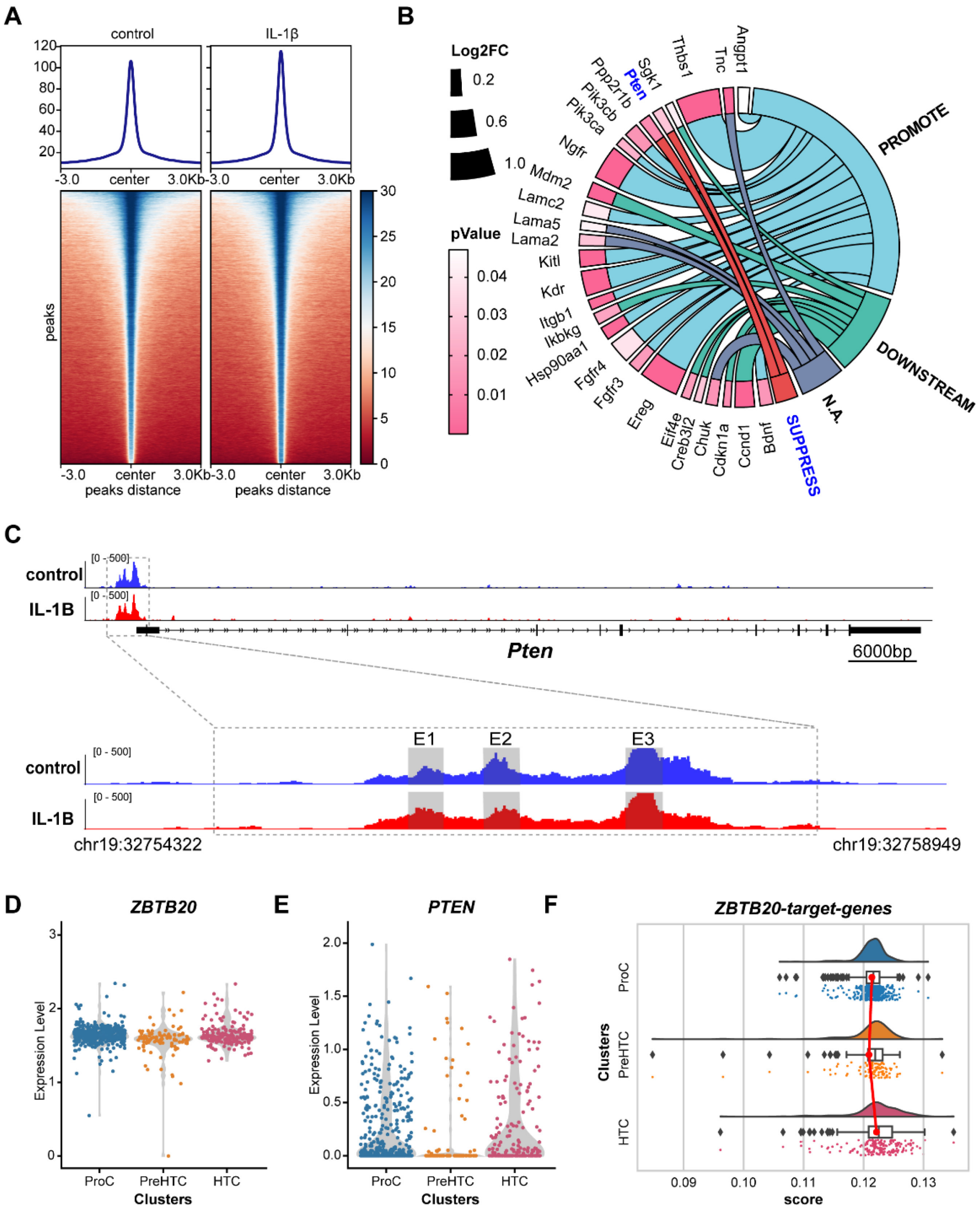

Fig. S6. Analysis of the transcription profile of ZBTB20 targets (**A**) Average profile (top) and heatmap (bottom) of ZBTB20 CUT&Tag-seq reads density around the center of peaks for the various conditions. (**B**) Chord plot shows a detailed relationship between the log2-fold change (log2FC) of DEGs (PI3K-Akt signaling associated genes with ZBTB20 occupation, left semicircle) and categories of their impact on signaling transduction (right semicircle). (**C**)

Representative tracks of CUT&Tag-seq analysis showing the enrichment of ZBTB20 around *Pten*'s promoter in chondrocytes. (**A**)-(**C**): n= 3 replicates. (**D-E**) Violin plots showing the expression distributions of *ZBTB20* (**D**), *PTEN* (**E**) in chondrocyte clusters (ProC, PreHTC, HTC). (**F**) Raincloud plot showing the expression distributions of ZBTB20-target-genes evaluated by AUCell score in certain cell clusters.

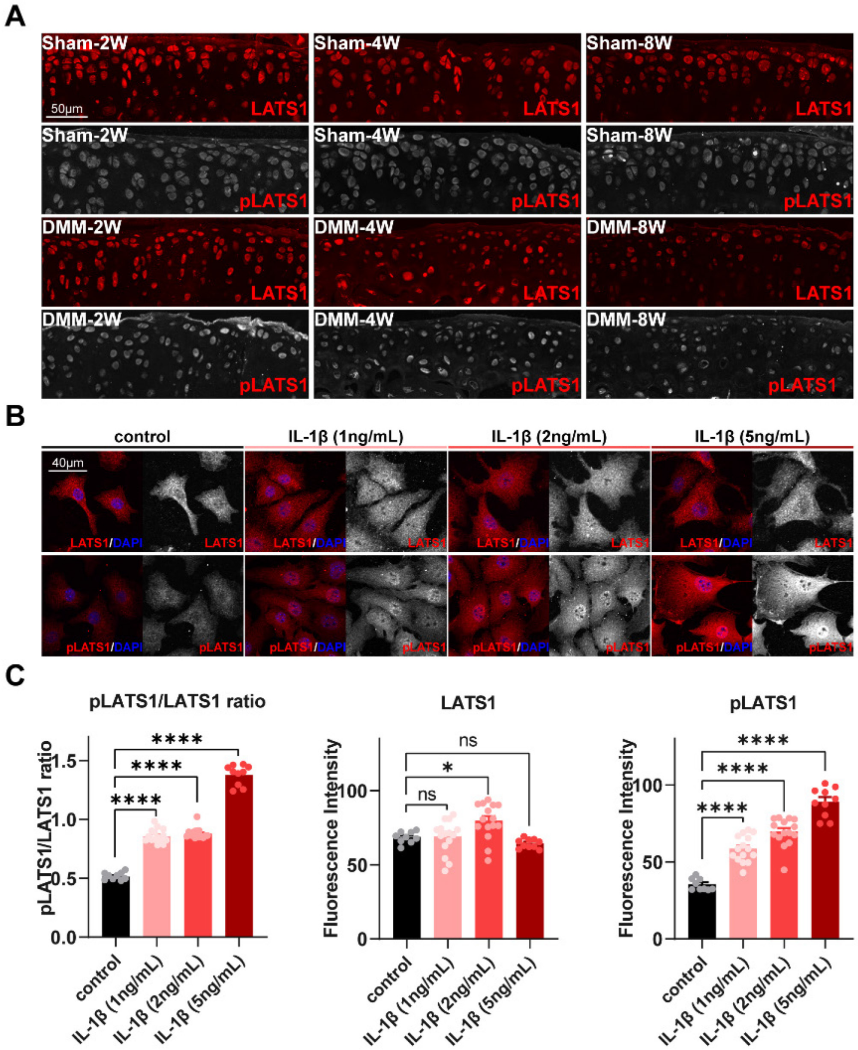

Fig. S7. Phosphorylation of LATS1 increases in OA chondrocytes (**A**) Representative images of immunofluorescence staining of LATS1, and pLATS1 in articular cartilage from mice underwent Sham or DMM surgery for 2/4/8W. n= 3 mice per group. (**B**) Representative images of immunofluorescence staining of LATS1, and pLATS1 in chondrocytes exposed to IL-1β ranging from 1 to 5 ng/mL. n= 3 biological independent experiments. (**C**) Statistical analysis of fluorescence signal of LATS1 and pLATS1 in indicated groups of cells in (**B**). n= 9, 15, 15, 10 views for one biological replicate.

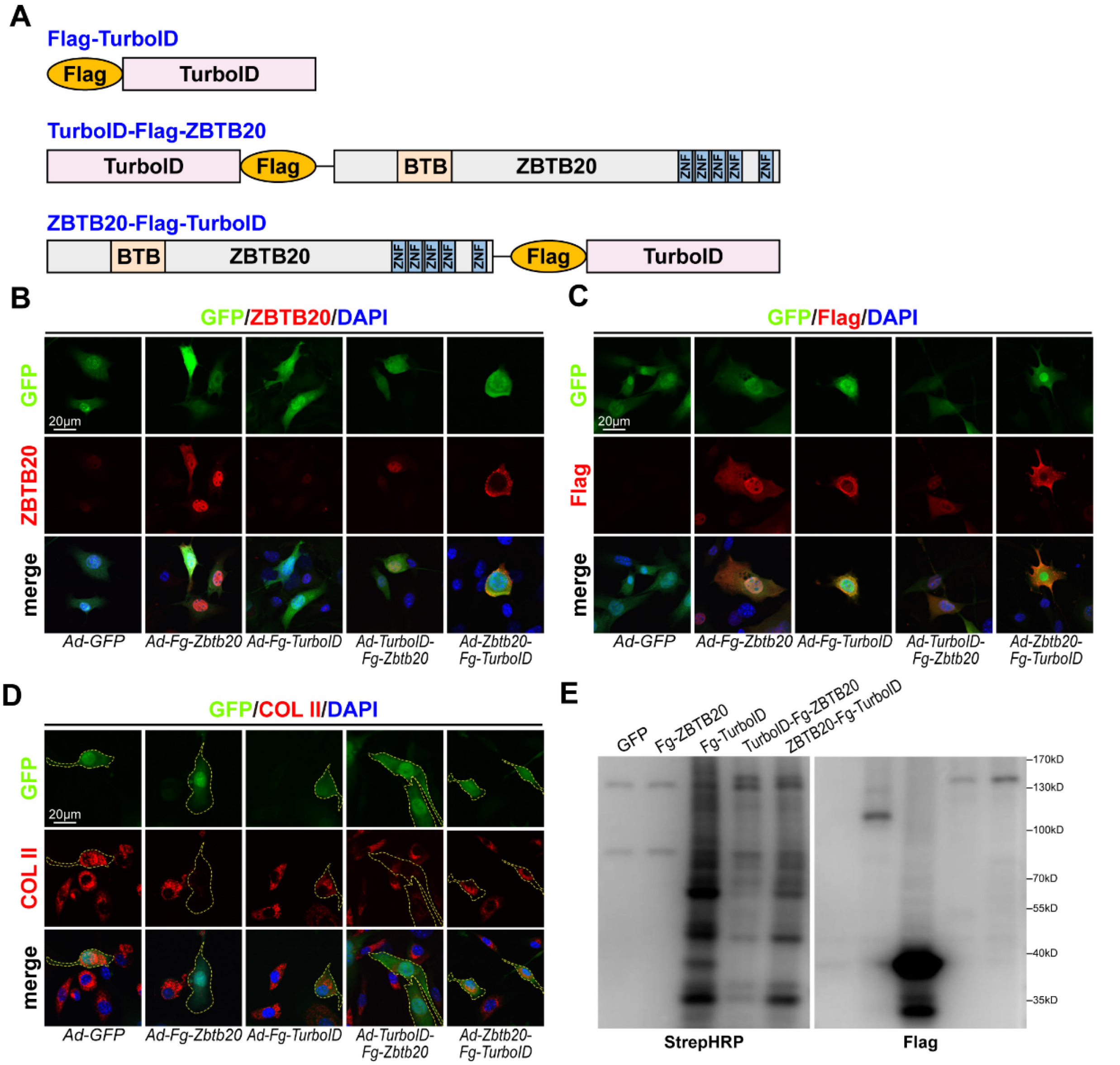

Fig. S8. Proximity labeling with TurboID and TurboID-ZBTB20 (**A**) Schematic diagrams of constructs of Flag-TurboID, TurboID-Flag-ZBTB20, ZBTB20-Flag-TurboID. (**B-D**) Representative images of immunofluorescence signal of ZBTB20 (**B**), Flag (**C**), and COL II (**D**) in chondrocytes transduced with indicated adenovirus. (**E**) Western blot of Biotin-labeled protein (by StrepHRP) and Flag in chondrocytes transduced with indicated adenovirus. (**B**)-(**E**): n= 3 biological independent experiments.

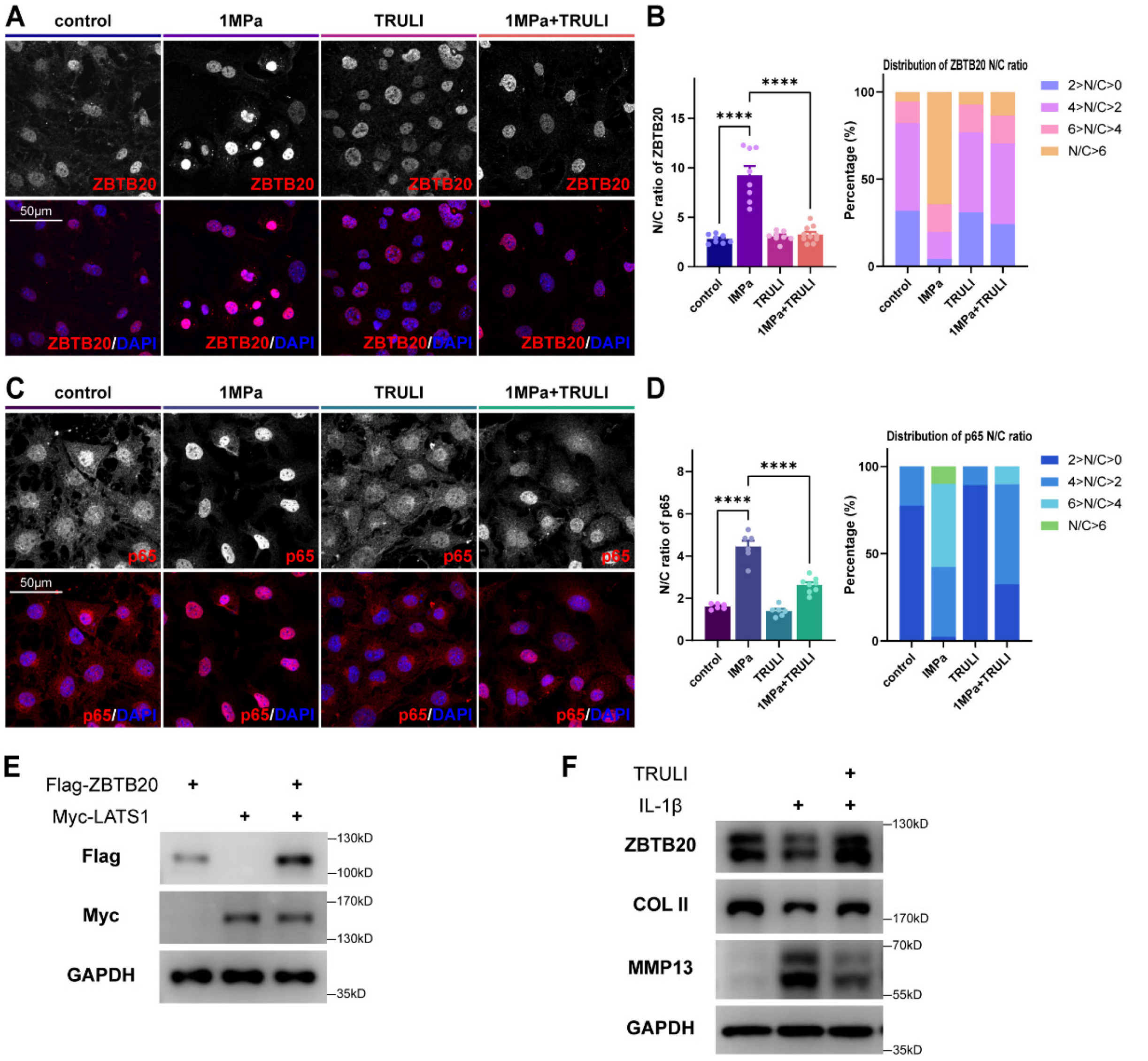

Fig. S9. The interaction between LATS1 and ZBTB20 could be influenced by mechanical loading, affecting the protein stability of ZBTB20. (**A**) Representative images of immunofluorescence staining of ZBTB20 in primary chondrocytes cultured at 1MPa with or without TRULI treatment. n= 3 biological independent experiments. (**B**) Statistical analysis of the cellular distribution of ZBTB20 in indicated groups of cells in (**A**). n= 8, 8, 8, 10 views per group for one biological replicate. The left panel is quantification of N/C ratio of ZBTB20. The right panel is the statistical analysis for ZBTB20 cellular distribution. (**C**) Representative images of immunofluorescence staining of p65 in primary chondrocytes cultured at 1MPa with or without TRULI treatment. n= 3 biological independent experiments. (**D**) Statistical analysis of the cellular distribution of p65 in indicated groups of cells in (**C**). n= 6, 6, 6, 8 views per group for one biological replicate. The left panel is quantification of N/C ratio of p65. The right panel is the statistical analysis for p65 cellular distribution. (**E**) Western blot of Flag, Myc, and

GAPDH in chondrocytes transfected with indicated *Ad-V*. (**F**) Western blot of ZBTB20, COL II, MMP13, and GAPDH in chondrocytes treated with IL-1β and TRULI.

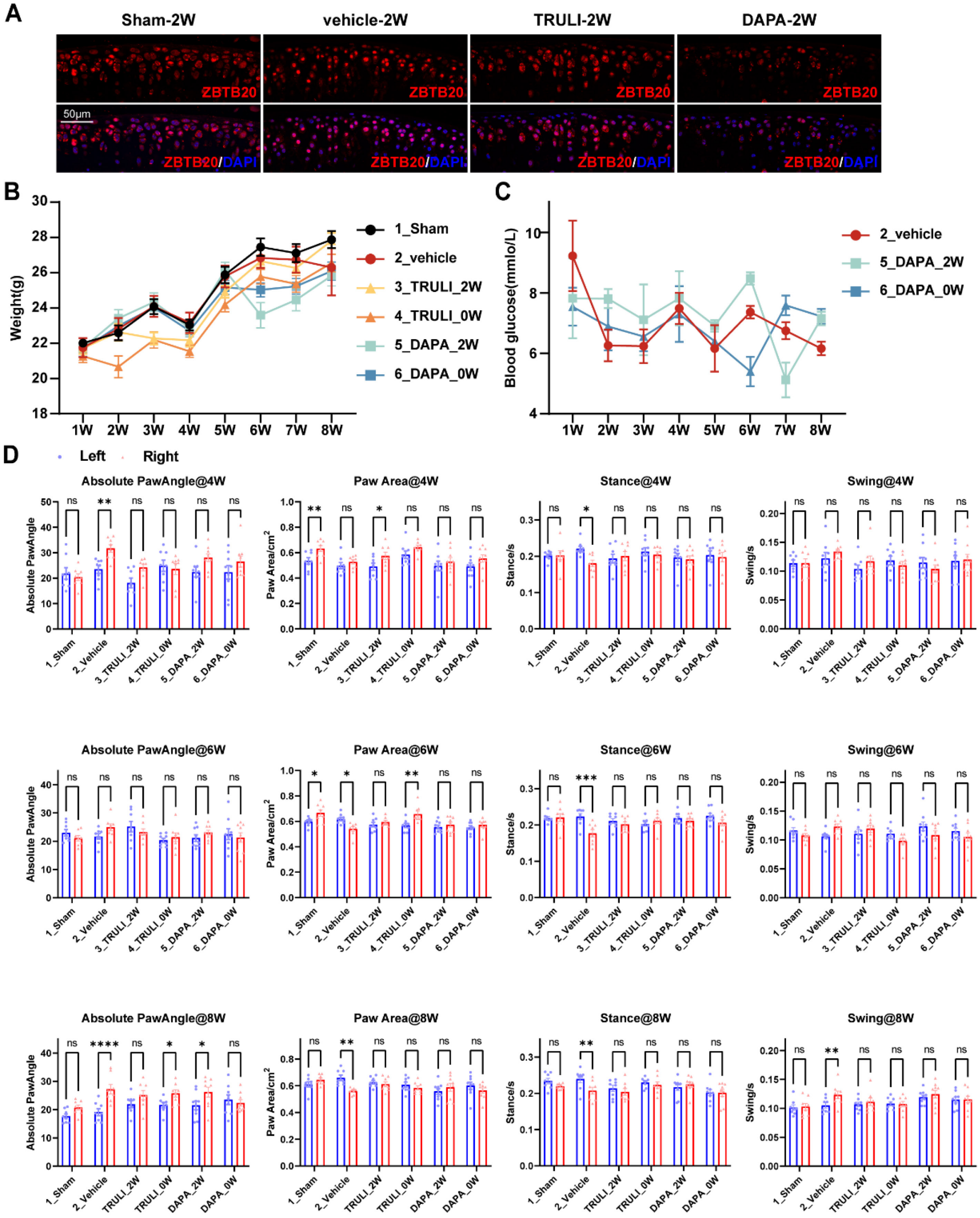

Fig. S10. Administration of TRULI and DAPA improves the abnormal behavior of OA in mice (**A**) IF staining of ZBTB20 in the tibial cartilage from indicated group of mice. (**B**) Curves of body weight of mice in indicated groups. n= 8, 8, 8, 8, 9, 9 mice. (**C**) Curves of blood glucose

of mice in indicated groups. n= 8, 9, 9 mice. (**D**) Statistical analysis of the Absolute PawAngle, Paw Area, Stance, and Swing of mice at 4/6/8 weeks post-surgery. n= 8, 8, 8, 8, 9, 9 mice.

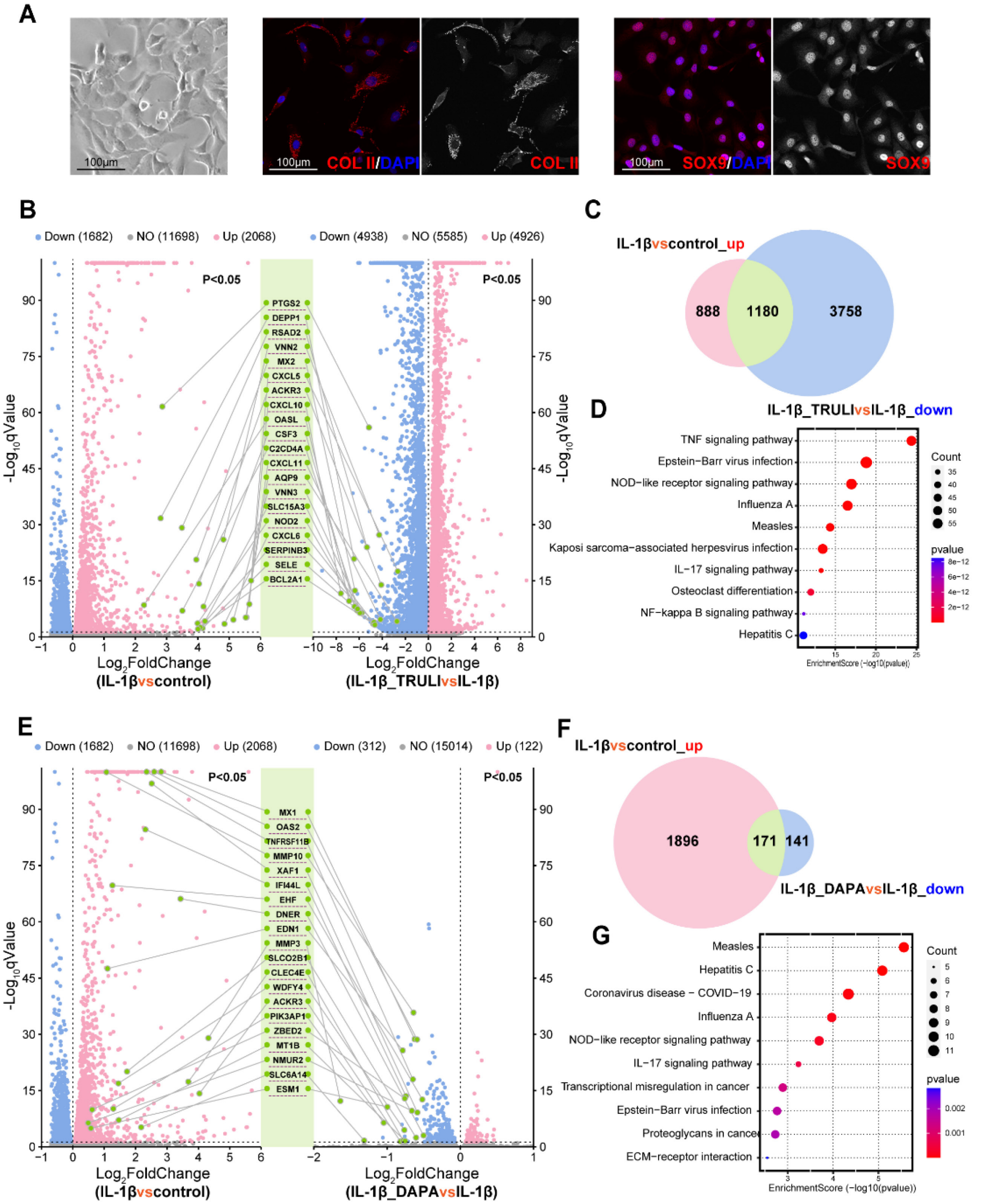

Fig. S11. Treatment of TRULI and DAPA alleviates inflammation in human primary chondrocytes (**A**) Representative images of brightfield and immunofluorescence staining of COL II, and SOX9 of the cultured human primary chondrocytes. (**B**) Double volcano graph showing the representative genes upregulated in comparison of IL-1β to control and downregulated in comparison of IL-1β_TRULI to IL-1β. (**C**) Venn diagram showing the numbers of DEGs of comparisons described in (**B**). (**D**) Dot plot graph of the enriched KEGG pathways of the overlapped DEGs in (**C**). (**E**) Double volcano graph showing the representative

genes upregulated in comparison of IL-1β to control and downregulated in comparison of IL-1β_DAPA to IL-1β. (**F**) Venn diagram showing the numbers of DEGs of comparisons described in (**E**). (**G**) Dot plot graph of the enriched KEGG pathways of the overlapped DEGs in (**E**). (**B**)-(**G**): n= 4 replicates.

Table S1. siRNA information

No.	Gene	Sequence
1	Mouse NC	TTCTCCGAACGTGTCACGT
2	Mouse *Zbtb20*-1	GAATCTACTCCGCACTCTA
3	Mouse *Zbtb20*-2	GUCAGUAACAGCUCCGAUAUU

Table S2. Antibody information

Antibody	Company	Catalog	Application/Dilution
ZBTB20	Proteintech	23987-1-AP	IHC (1:80); IF (1:100); WB (1:1000)
ZBTB20	Abcam	ab243143	WB (1:1000)
COL II	Thermo	MA5-13026	IHC (1:100); IF (1:100)
COL II	Proteintech	28459-1-AP	WB (1:1000)
LATS1	Cell Signaling	3477	IF (1:100); WB (1:1000)
pLATS1-T1079	Cell Signaling	8654	IF (1:100); WB (1:1000)
ACAN	Sigma	AB1031	IF (1:300)
MMP13	Proteintech	18165-1-AP	IF (1:100); WB (1:1000)
ADAMTS5	Abcam	ab41037	IF (1:100)
ADAMTS5	BOSTER	A02802-1	WB (1:1000)
p65	Invitrogen	51-0500	IF (1:100)
p65	Cell Signaling	8242	WB (1:1000)
FLAG	Sigma	F3165-.2MG	IF (1:100); WB (1:1000); IP (2μg per IP)
p65	Cell Signaling	14946	WB (1:1000)
SOX9	Abcam	ab185966	IF (1:100)
GAPDH	Proteintech	60004-1-Ig	WB (1:1000)
AKT	Cell Signaling	4685	WB (1:1000)
pAKT-T308	Cell Signaling	13038	WB (1:1000)
pAKT-S473	Cell Signaling	4060	WB (1:1000)
pp65-S536	Cell Signaling	3033	WB (1:1000)
β-Tublin	ABclonal	AC021	WB (1:1000)
LaminB1	ABclonal	A1910	WB (1:1000)
MYC	Smart-Lifesciences	SLAB2901	WB (1:1000); IP (2μg per IP)
PTEN	Cell Signaling	9188	WB (1:1000)
HRP-conjugated Streptavidin	Proteintech	SA00001-0	WB (1:1000)

Table S3. Primer information

No.	Name	5'-3' Sequence	Note
1	*Gapdh*-F	AGGTCGGTGTGAACGGATTTG	qPCR
	Gapdh-R	TGTAGACCATGTAGTTGAGGTCA	qPCR
2	*Zbtb20*-F	AACGCAATGAATCCGAGGAGT	qPCR
	Zbtb20-R	CCCAAACTGTTGCTCCACTGA	qPCR
3	*Nos2*-F	CTATCAGGAAGAAATGCAGGAGAT	qPCR
	Nos2-R	GAGCACGCTGAGTACCTCATT	qPCR
4	*Col2a1*-F	CACCAAATTCCTGTTCAGCC	qPCR
	Col2a1-R	TGCACGAAACACACTGGTAAG	qPCR
5	*Acan*-F	GGAGCGAGGCCATTTACAAC	qPCR
	Acan-R	CGTAGACAAGGTAGCCCACTTT	qPCR
6	*Mmp3*-F	TGGACAGAGGATGTCACTGGTA	qPCR
	Mmp3-R	GCCTTGGCTGAGTGGTAGAG	qPCR
7	*Adamts5*-F	GGAGCGAGGCCATTTACAAC	qPCR
	Adamts5-R	CGTAGACAAGGTAGCCCACTTT	qPCR
8	*Mmp13*-F	CTTCTTCTTGTTGAGCTGGACTC	qPCR
	Mmp13-R	CTGTGGAGGTCACTGTAGACT	qPCR
9	*Pten*-F	TGAGTTCCCTCAGCCATTGCCT	qPCR
	Pten-R	GAGGTTTCCTCTGGTCCTGGTA	qPCR
10	*Pten*-1-F	CAGACCACACGGGAGAAGGA	ChIP-qPCR
	Pten-1-R	GGCTCAAAGGCGAGGTGGA	ChIP-qPCR
11	*Pten*-2-F	CGCTGGCTTAGCTTGGGC	ChIP-qPCR
	Pten-2-R	CTCAGAGAGCTATGGGC	ChIP-qPCR

Table S4. Genotyping primer information

No.	Name	5'-3' Sequence
1	*Col2a1-CreER*^{T2}-F	ATCCGAAAAGAAAACGTTGA
2	*Col2a1-CreER*^{T2}-R	ATCCAGGTTACGGATATAGT
3	*Zbtb20-floxed*-P1	GCACCGCTGGCAACACCTATCTG
4	*Zbtb20-floxed*-P2	CTCTCCCCTCCTCCCTCTGG
